# RGS16 Aggravates Hepatic Ischemia‐Reperfusion Injury via Hepatocyte‐Intrinsic Apoptosis/Inflammation & Neutrophil Recruitment/NETosis

**DOI:** 10.1002/advs.76817

**Published:** 2026-07-28

**Authors:** Xinglong Li, Zhanzhi Meng, Hongjun Yu, Yongliang Hua, Zihao Li, Bing Yin, Baolin Qian, Zhongyu Li, Yongzhi Zhou, Zhigang Feng, Shounan Lu, Shanjia Ke, Miaoyu Bai, Yao Fu, Wei Tang, Natalia V. Belosludtseva, Yong Ma

**Affiliations:** ^1^ Department of Minimally Invasive Hepatic Surgery The First Affiliated Hospital of Harbin Medical University Harbin China; ^2^ Key Laboratory of Hepatosplenic Surgery Ministry of Education The First Affiliated Hospital of Harbin Medical University Harbin China; ^3^ Department of General Surgery The Fourth Affiliated Hospital of Harbin Medical University Harbin China; ^4^ Department of Pediatric Surgery The Sixth Affiliated Hospital of Harbin Medical University Harbin China; ^5^ Department of Hepatobiliary Surgery Affiliated Hospital of Southwest Medical University Luzhou China; ^6^ The First Department of General Surgery Affiliated Hospital of Inner Mongolia Minzu University Tongliao China; ^7^ Department of Ultrasound The First Affiliated Hospital of Harbin Medical University Harbin China; ^8^ International Health Care Center National Center for Global Health and Medicine Tokyo Japan; ^9^ Hepato‐Biliary‐Pancreatic Surgery Division Department of Surgery The University of Tokyo Hospital Tokyo Japan; ^10^ Institute of Theoretical and Experimental Biophysics Russian Academy of Sciences Pushchino Russia

**Keywords:** apoptosis, hepatic ischemia‒reperfusion injury, inflammation, neutrophil extracellular traps, regulator of G‐protein signaling 16

## Abstract

Hepatic ischemia–reperfusion injury (HIRI) is a frequent and severe complication after liver transplantation and hepatectomy, driven by sterile inflammation and lacking effective therapies. Regulator of G‐protein signaling 16 (RGS16) is an inflammatory modulator, but its role in HIRI remains unclear. We found that RGS16 expression was markedly increased in the livers of post‐hepatectomy patients and positively correlated with neutrophil extracellular trap formation (NETosis). Using hepatocyte‐specific *Rgs16* knockout and transgenic mouse models, we demonstrated that Rgs16 deficiency attenuated hepatic inflammation and apoptosis after HIRI, whereas Rgs16 overexpression significantly exacerbated liver injury. Integrated multi‐omics analyses, including RNA‐seq, MeRIP‐seq, and mass spectrometry, identified C‐X‐C motif chemokine ligand 1 (CXCL1) as a key downstream effector of RGS16. Mechanistically, RGS16 competitively binds YTH domain family protein 3 (YTHDF3), displacing poly(A)‐nuclease deadenylation complex subunit 3 (PAN3) and stabilizing m^6^A‐modified Cxcl1 mRNA, thereby enhancing CXCL1 expression. Elevated CXCL1 promotes hepatocyte apoptosis and inflammation through C‐X‐C chemokine receptor 2 (CXCR2) and drives neutrophil recruitment and NETosis. Pharmacological inhibition of CXCL1 or degradation of NETs with DNase I significantly alleviated RGS16‐induced liver injury. Collectively, RGS16 aggravates HIRI by amplifying hepatocyte‐intrinsic CXCL1–CXCR2 signaling and CXCL1‐mediated neutrophil recruitment and NETosis, suggesting RGS16 and its downstream pathways as potential therapeutic targets for HIRI.

## Graphical abstract

RGS16 competitively interferes with the YTHDF3–PAN3 complex to prevent CXCL1 mRNA decay during hepatic ischemia‐reperfusion injury. Stabilized CXCL1 strengthens hepatocyte injury, neutrophil recruitment, and NET‐associated inflammation, uncovering how stress‐induced RGS16 converts post‐transcriptional regulation into immune‐mediated liver damage.

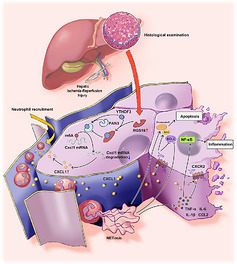

## 1 Introduction

Hepatic ischemia‒reperfusion injury (HIRI) is an inevitable consequence of liver transplantation or resection, driven by cellular death, oxidative stress, and inflammatory immune reactions [1, 2, 3]. While the liver tolerates ischemia‐induced oxygen deprivation and ATP insufficiency, reperfusion triggers intense oxidative stress and inflammation, leading to hepatocyte damage and necrosis [4]. This assertion is supported by a multitude of prior studies conducted by our research team and others [5, 6, 7]. These pathological processes create a harmful cycle, resulting in severe or irreversible liver dysfunction. Disrupting this cycle could mitigate liver damage and improve surgical outcomes.

Regulator of G‐protein signaling 16 (RGS16), highly expressed in the liver, plays a significant role in cellular signaling and inflammation [8]. Hepatocyte‐specific overexpression of RGS16 in transgenic mice predisposes them to fatty liver under a high‐fat diet, while Rgs16 knockdown alleviates hepatic steatosis and inflammation [9, 10]. Additionally, RGS16 has been implicated in adaptive immunity, where it influences allergic inflammation by modulating chemokine receptors, such as CXCR4, CCR3, and CCR5, which are critical for T cell migration and activation [11]. However, its role in HIRI remains unclear. Given its involvement in inflammation and liver diseases, we hypothesized that RGS16 may function as a stress‐inducible mediator that contributes to HIRI progression.

C‐X‐C motif chemokine ligand 1 (CXCL1), a key chemokine in inflammatory states, is significantly upregulated in HIRI, promoting neutrophil infiltration, and activation [12]. Activated neutrophils release neutrophil extracellular traps (NETs) through NETosis, and dysregulation of this process exacerbates tissue damage, contributing to HIRI [13, 14].

In the present study, we observed a noteworthy increase in the expression of RGS16 during HIRI. Both in vivo and in vitro studies demonstrated that RGS16 exacerbated HIRI by upregulating CXCL1 in hepatocytes, leading to hepatocyte apoptosis, inflammation, and neutrophil recruitment. Subsequent NETosis further amplified the inflammatory response and cell apoptosis, aggravating liver damage. Mechanistically, RGS16 promoted CXCL1 expression by binding to YTH domain family protein 3 (YTHDF3) and further competitively inhibited the binding of YTHDF3 to poly(A)‐nuclease deadenylation complex subunit 3 (PAN3). This reduced the YTHDF3‐mediated degradation of N6‐methyladenosine (m^6^A)‐modified CXCL1, increasing its stability and promoting hepatocyte apoptosis, inflammation, neutrophil recruitment, and NETosis. These findings highlight RGS16 as a stress‐inducible functional pathogenic amplifier of HIRI through CXCL1, suggesting that targeting RGS16 and its pathway may offer a novel therapeutic strategy for HIRI.

## 2 Results

### 2.1 Increased RGS16 Expression is Correlated With Clinical Outcomes in HIRI

**FIGURE 1:**
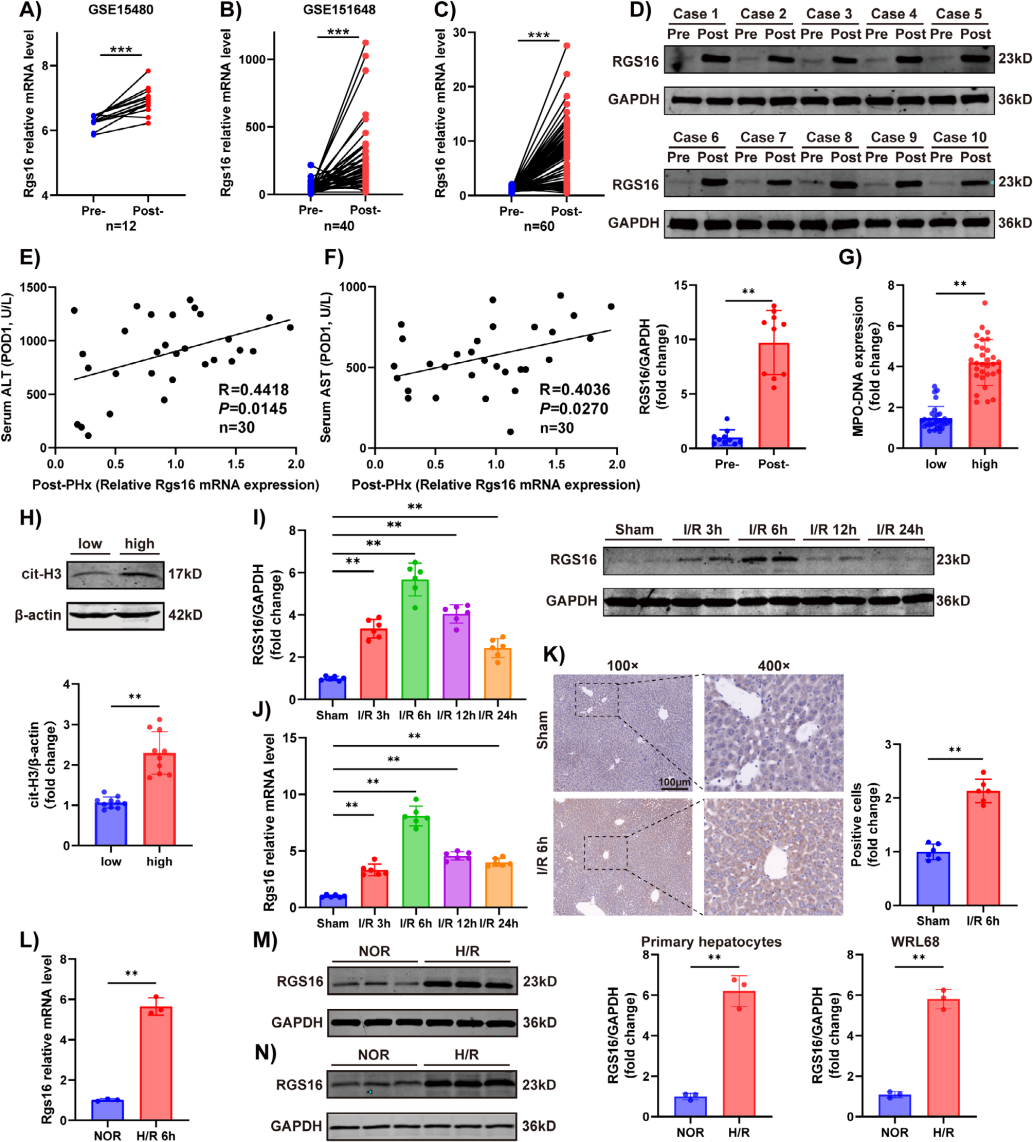
RGS16 expression is increased in HIRI. (A, B) RGS16 expression was analyzed based on the GEO datasets GSE15480 (*n* = 12 pairs) and GSE151648 (*n* = 40 pairs), and the samples were divided into pre‐ and post‐ischemia groups. (C) RGS16 mRNA levels in liver samples from patients who underwent partial hepatectomy (*n* = 60). (D) Protein levels of RGS16 in liver samples from patients who underwent partial hepatectomy (*n* = 10). (E, F) Correlation analysis between the RGS16/GAPDH ratio post‐hepatectomy and serum ALT/AST levels at POD1 (*n* = 30). (G) Comparison of the ratio of MPO‐DNA content in liver tissues between the RGS16 high‐expression group (*n* = 30) and low‐expression group (*n* = 30) before and after surgery. (H) Western blot analysis of cit‐H3 in liver tissues from the RGS16 high‐expression group (*n* = 10) and low‐expression group (*n* = 10) after surgery. (I, J) RGS16 protein levels (I) and mRNA expression (J) in the livers of WT mice subjected to 1.5 h of ischemia followed by reperfusion for 3, 6, 12, or 24 h; *n* = 6 per time point. (K) IHC staining of RGS16 in liver sections from WT mice after HIRI (*n* = 6). Scale bar = 100 µm. (L, M) Rgs16 mRNA levels and protein expression in hepatocytes isolated from mouse livers subjected to H/R challenge (6/6 h) (*n* = 3). (N) RGS16 protein expression in WRL68 cells subjected to H/R challenge (6/6 h) (*n* = 3). Data were normalized to GAPDH or β‐actin and are presented as mean ± SD. Two‐tailed paired Student's *t*‐test was used for paired pre‐ and post‐surgery comparisons. Two‐tailed unpaired Student's *t*‐test was used for comparisons between two independent groups. One‐way ANOVA followed by Tukey's multiple comparison test was used for comparisons among multiple groups. Pearson's correlation analysis was used for correlation analysis. **p < 0.01, ***p < 0.001, n.s., not significant.

Bioinformatics analysis of the GSE15480 (*n* = 12) and GSE151648 (*n* = 40) datasets revealed significantly elevated RGS16 expression in liver tissues post‐transplantation (*p* = 7.01 × 10^−^^7^ and *p* = 9.7 × 10^−^^4^, respectively) (Figure 1A,B). To explore the association between RGS16 expression and HIRI, we analyzed liver samples from patients undergoing partial hepatectomy (*n* = 60) (Figure 1C,D). Notably, the positive correlations between postoperative Rgs16 mRNA expression levels and serum alanine aminotransferase (ALT) (R = 0.4418, *p* = 0.0145) and aspartate aminotransferase (AST) (R = 0.4036, *p* = 0.0270) levels on postoperative day 1 (POD1) indicated that higher RGS16 expression is associated with more severe liver damage (Figure 1E,F). Given the role of NETs in HIRI, we investigated the correlation between RGS16 and NETs. Patients were divided into high‐ (*n* = 30) and low‐RGS16 expression groups (*n* = 30) based on the median postoperative/preoperative RGS16 ratios. The high‐RGS16 group exhibited significantly greater myeloperoxidase‐DNA (MPO‐DNA) and citrullinated histone H3 (cit‐H3) levels, indicating a strong RGS16‐NETs association (Figure 1G,H). In a murine HIRI model, Rgs16 mRNA and protein levels were significantly elevated in the liver compared to sham controls (Figure 1I,J), with immunohistochemistry (IHC) confirming increased hepatocyte RGS16 expression (Figure 1K). Similarly, hypoxia/reoxygenation (H/R) stimulation upregulated RGS16 expression in both primary hepatocytes and WRL68 cells, suggesting hepatocytes as key target cells of RGS16 (Figure 1L–N). These findings indicate that increased RGS16 expression post‐HIRI is associated with poor outcomes and may function as a pathogenic mediator in HIRI pathogenesis, partially through NETs.

### 2.2 RGS16 Accelerates the Hepatocyte Inflammatory Response and Cell Apoptosis During H/R Injury

**FIGURE 2:**
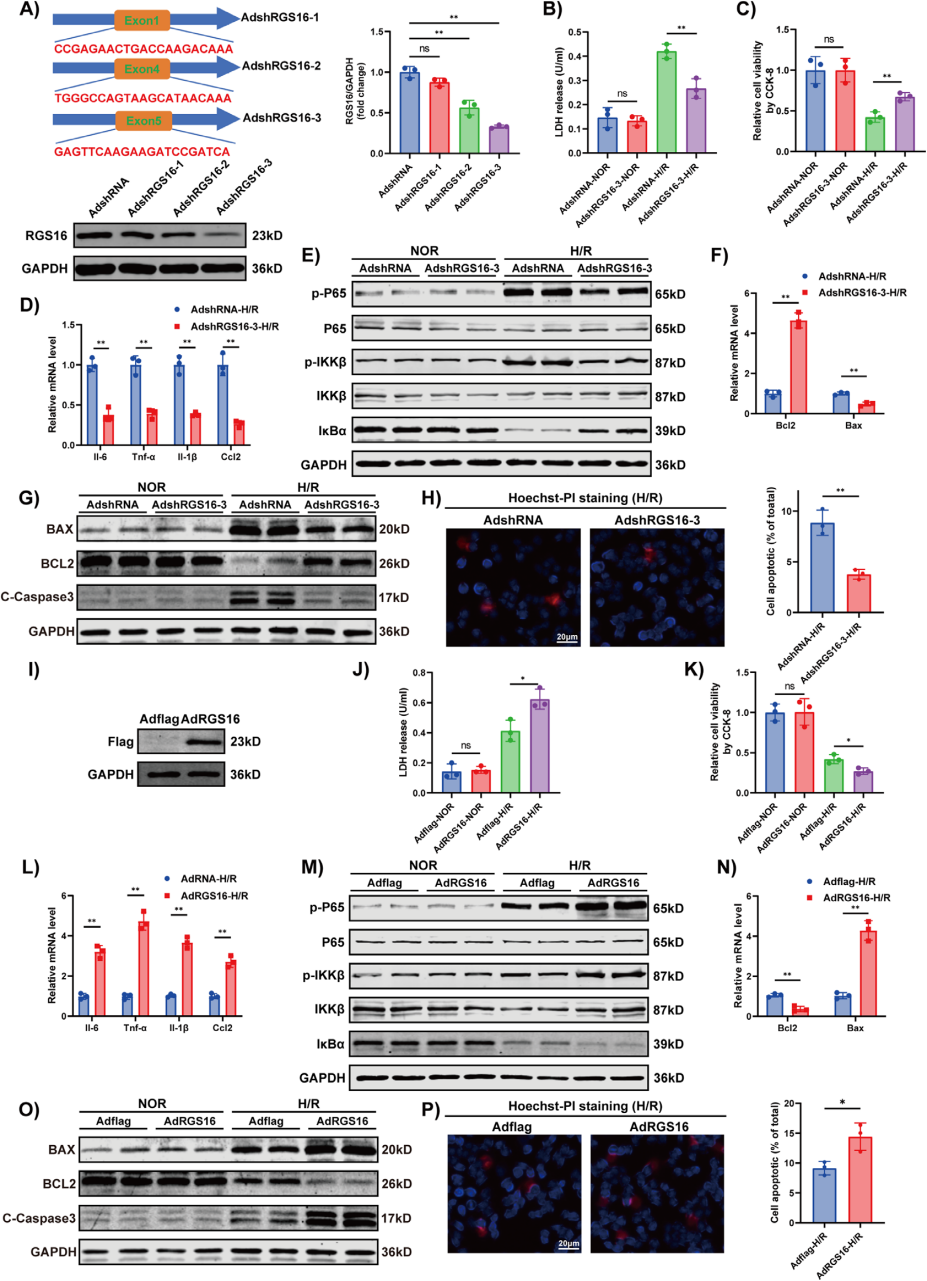
RGS16 exacerbates hepatic H/R injury. (A) Schematic diagram of the workflow and western blot analysis showing the transfection efficiency of RGS16 knockdown (*n* = 3). (B) LDH assay showing cell damage (*n* = 3). (C) CCK‐8 assay showing cell viability (*n* = 3). (D) qRT‐PCR analysis of proinflammatory factor mRNA levels (*n* = 3). (E) Western blot analysis of NF‐κB signaling pathway molecules (*n* = 3). (F) Relative mRNA expression levels of Bcl2 and Bax detected by qRT‐PCR (*n* = 3). (G) Western blot analysis of BCL2, BAX, and cleaved caspase‐3 protein levels (*n* = 3). (H) Representative Hoechst/PI staining showing apoptotic cells (*n* = 3). Scale bar = 20 µm. (I) Western blot analysis showing RGS16 overexpression efficiency (*n* = 3). (J) LDH assay showing cell damage (*n* = 3). (K) CCK‐8 assay showing cell viability (*n* = 3). (L) qRT‐PCR analysis of proinflammatory factor mRNA levels (*n* = 3). (M) Western blot analysis of NF‐κB signaling pathway molecules (*n* = 3). (N) Relative mRNA expression levels of Bcl2 and Bax detected by qRT‐PCR (*n* = 3). (O) Western blot analysis of BCL2, BAX, and cleaved caspase‐3 protein levels (*n* = 3). (P) Representative Hoechst/PI staining showing apoptotic cells (*n* = 3). Scale bar = 20 µm. Data were normalized to GAPDH and are presented as mean ± SD. Two‐tailed unpaired Student's *t*‐test was used for comparisons between two groups. One‐way ANOVA followed by Tukey's multiple comparison test was used for comparisons among multiple groups. *p < 0.05, **p < 0.01, n.s., not significant.

To evaluate the function of RGS16 in hepatocytes in response to H/R challenge, we established three *Rgs16*‐knockdown adenoviral vectors and verified their knockdown efficiency (Figure 2A). Primary hepatocytes were subsequently examined for cell viability, lactate dehydrogenase (LDH) levels, inflammation, and cell apoptosis‐related pathways after hypoxia for 6 h followed by reoxygenation for 6 h. Compared to the AdshRNA control, *Rgs16* knockdown significantly reduced LDH levels, increased cell viability, and attenuated inflammation after H/R challenge (Figure 2B–E and Figure S1A). Additionally, *Rgs16* knockdown upregulated the mRNA and protein levels of the antiapoptotic factor B‐cell lymphoma 2 (BCL2), while downregulating the proapoptotic factors Bcl2‐associated X (BAX) and cleaved‐caspase3 (C‐Caspase3) (Figure 2F,G and Figure S1B). Hoechst‐PI staining revealed that *Rgs16* knockdown significantly alleviated apoptosis in H/R‐treated hepatocytes (Figure 2H). Conversely, we designed an *Rgs16*‐overexpressing adenoviral vector and verified its overexpression efficiency (Figure 2I). *Rgs16* overexpression exacerbated H/R‐induced cell damage, inflammation, and apoptosis (Figure 2J–P and Figure S1C,D). These findings suggest that RGS16 accelerates H/R‐induced inflammation and apoptosis in hepatocytes.

### 2.3 Hepatocyte‐Specific Rgs16 Deficiency Alleviates the Liver Inflammatory Response and Apoptosis During HIRI

**FIGURE 3:**
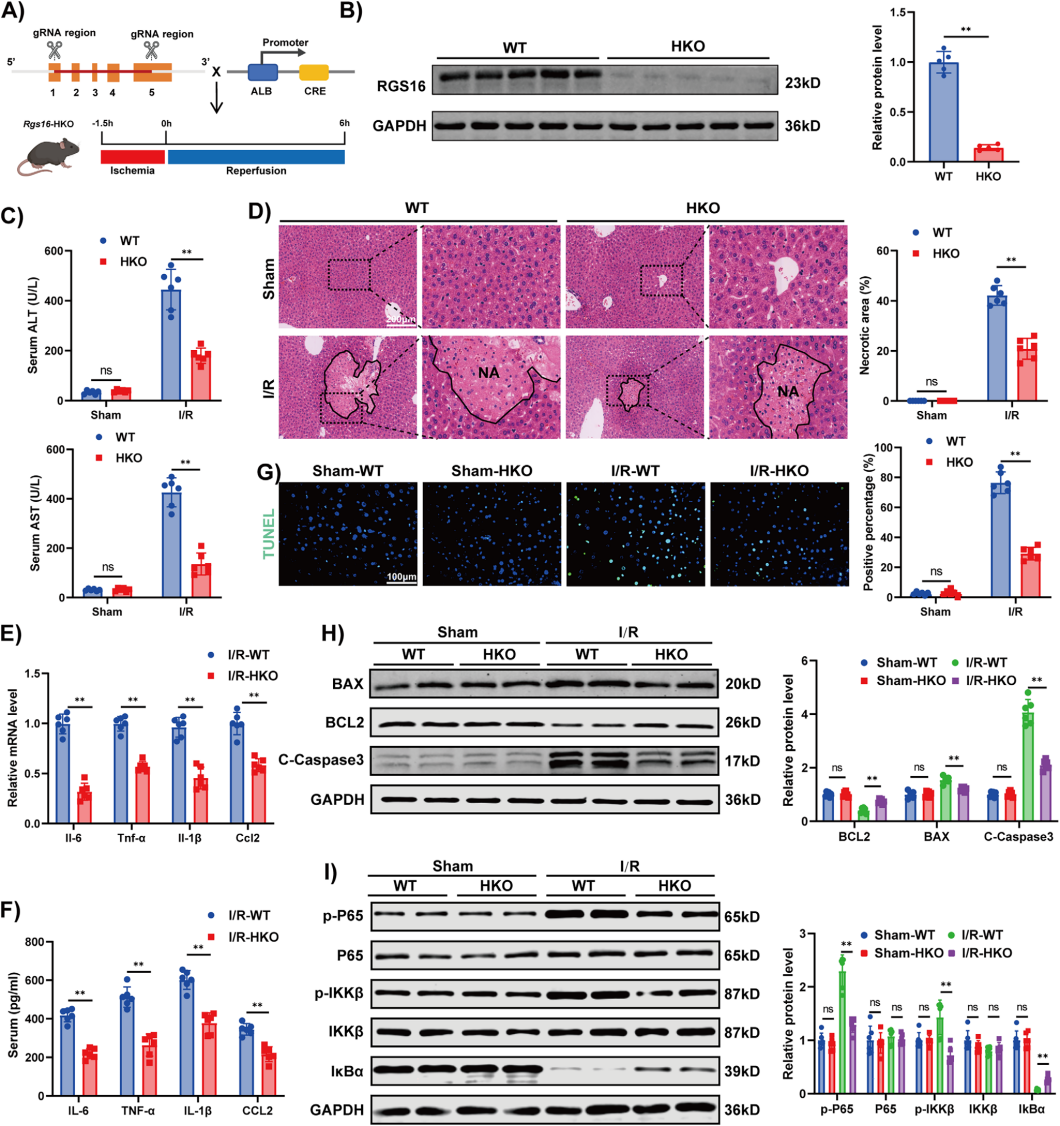
Hepatocyte‐specific RGS16 deficiency alleviates liver inflammatory responses and apoptosis during HIRI. (A) Schematic diagram showing the generation of *Rgs16*‐HKO mice. (B) RGS16 deletion efficiency was measured by western blot analysis (*n* = 5). (C) Serum ALT and AST levels in WT and *Rgs16*‐HKO mice after HIRI (*n* = 6). (D) H&E staining of liver sections from WT and *Rgs16*‐HKO mice after HIRI (*n* = 6). Scale bar = 200 µm. NA, necrotic area. (E) Relative mRNA expression levels of proinflammatory factors in liver tissues from WT and *Rgs16*‐HKO mice after HIRI (*n* = 6). (F) Serum levels of proinflammatory factors in WT and *Rgs16*‐HKO mice after HIRI (*n* = 6). (G) TUNEL staining of liver sections from WT and *Rgs16*‐HKO mice after HIRI (*n* = 6). Scale bar = 100 µm. (H) Protein levels of BCL2, BAX, and cleaved caspase‐3 in liver tissues from WT and *Rgs16*‐HKO mice after HIRI (*n* = 6). (I) Protein levels of NF‐κB signaling pathway molecules in liver tissues from WT and *Rgs16*‐HKO mice after HIRI (*n* = 6). Data were normalized to GAPDH where applicable and are presented as mean ± SD. Two‐tailed unpaired Student's *t*‐test was used for comparisons between WT and *Rgs16*‐HKO groups. *p < 0.05, **p < 0.01, n.s., not significant.

To evaluate the functional role of RGS16 in HIRI in vivo, ALB‐Cre mice were bred with *Rgs16^f/f^* mice to generate *Rgs16*‐HKO and subjected to HIRI (Figure 3A). Successful knockout of *Rgs16* in the liver was confirmed via Western blot (Figure 3B). Serum AST and ALT levels and necrotic areas were assessed to evaluate the extent of liver damage. *Rgs16*‐HKO mice exhibited significantly lower serum ALT and AST levels at 6 h post‐I/R surgery compared to wild‐type (WT) mice (Figure 3C). In addition, the necrotic area was considerably smaller in liver sections from *Rgs16*‐HKO mice than in those from control mice, as assessed by H&E staining (Figure 3D). Consistent with the in vitro results, *Rgs16*‐HKO mice presented a reduced inflammatory response, as verified by ELISAs and the mRNA levels of Tumor Necrosis Factor‐alpha (TNFα), Interleukin‐6 (IL‐6), Interleukin‐1 beta (IL‐1β), and C─C Motif Chemokine Ligand 2 (CCl2) (Figure 3E,F). TUNEL staining demonstrated that *Rgs16*‐HKO mice had fewer apoptotic cells in the liver compared to WT mice after HIRI (Figure 3G). Furthermore, the protein expression levels of BAX and C‐Caspase3 were lower, whereas those of BCL‐2 were significantly higher in the *Rgs16*‐HKO mice than in the control mice (Figure 3H). Additionally, key marker proteins of the NF‐κB pathway indicated that RGS16 knockout in hepatocytes alleviated the inflammation response (Figure 3I). Taken together, these findings suggest that RGS16 knockout protects against I/R‐induced inflammation and apoptosis in liver tissue.

### 2.4 Hepatocyte‐Specific *Rgs16* Overexpression Exacerbates Liver Apoptosis and the Inflammatory Response During HIRI

**FIGURE 4:**
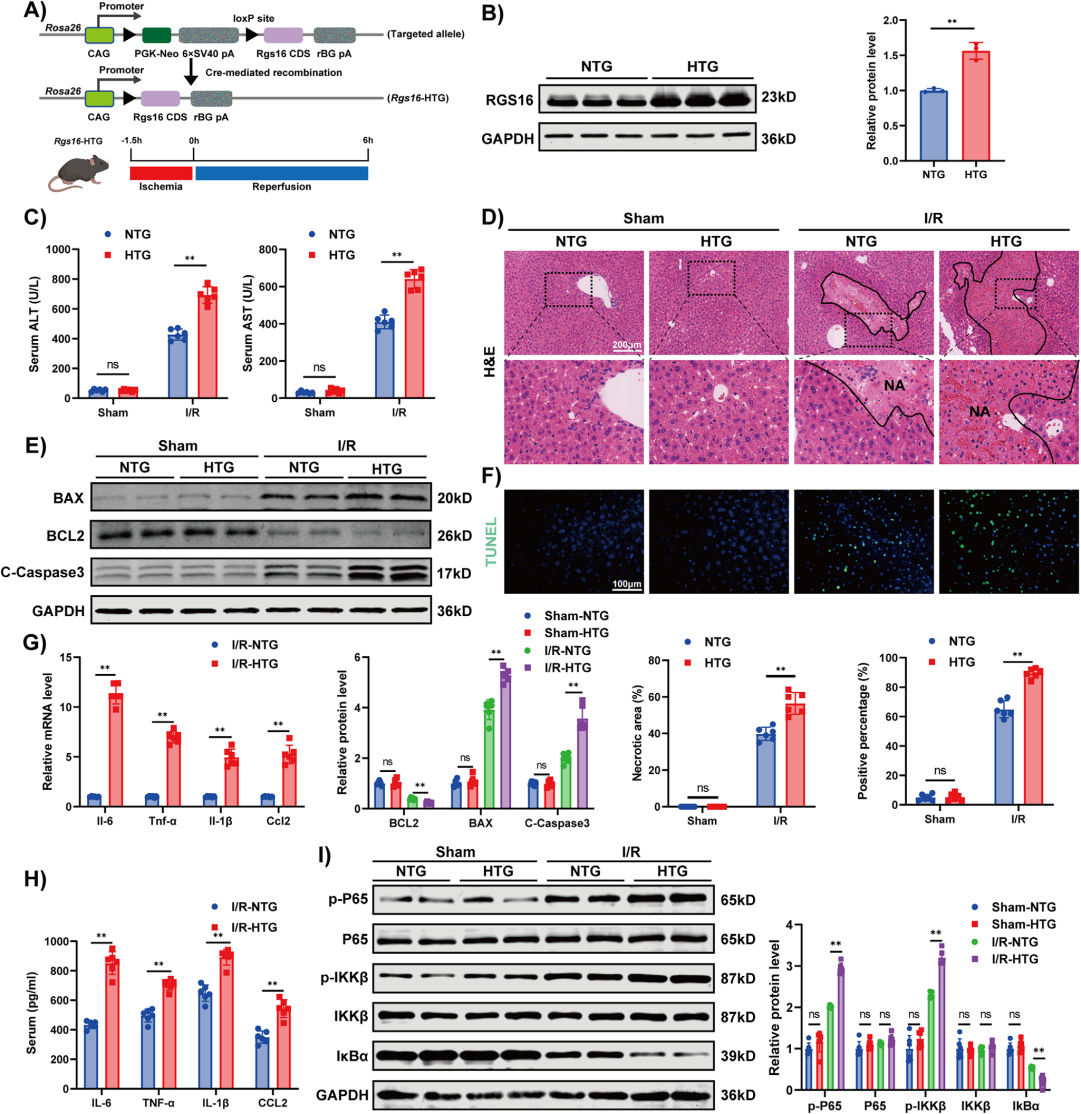
Hepatocyte‐specific RGS16 overexpression aggravates liver inflammatory responses and apoptosis during HIRI. (A) Schematic diagram showing the generation of *Rgs16*‐HTG mice. (B) RGS16 overexpression efficiency was measured by western blot analysis (*n* = 3). (C) Serum ALT and AST levels in *Rgs16*‐NTG and *Rgs16*‐HTG mice after HIRI (*n* = 6). (D) H&E staining of liver sections from *Rgs16*‐NTG and *Rgs16*‐HTG mice after HIRI (*n* = 6). Scale bar = 200 µm. NA, necrotic area. (E) Protein levels of BCL2, BAX, and cleaved caspase‐3 in liver tissues from *Rgs16*‐NTG and *Rgs16*‐HTG mice after HIRI (*n* = 6). (F) TUNEL staining of liver sections from *Rgs16*‐NTG and *Rgs16*‐HTG mice after HIRI (*n* = 6). Scale bar = 100 µm. (G) Relative mRNA expression levels of proinflammatory factors in liver tissues from *Rgs16*‐NTG and *Rgs16*‐HTG mice after HIRI (*n* = 6). (H) Serum levels of proinflammatory factors in *Rgs16*‐NTG and *Rgs16*‐HTG mice after HIRI (*n* = 6). (I) Protein levels of NF‐κB signaling pathway molecules in liver tissues from *Rgs16*‐NTG and *Rgs16*‐HTG mice after HIRI (*n* = 6). Data were normalized to GAPDH where applicable and are presented as mean ± SD. Two‐tailed unpaired Student's *t*‐test was used for comparisons between *Rgs16*‐NTG and *Rgs16*‐HTG groups. *p < 0.05, **p < 0.01, n.s., not significant.

To further assess the effect of RGS16 on HIRI, we generated *Rgs16*‐HTG mice (Figure 4A). Western blot analysis confirmed the successful overexpression of RGS16 (Figure 4B). In contrast to the findings in *Rgs16*‐HKO mice, in *Rgs16*‐HTG mice, RGS16 overexpression markedly elevated I/R‐induced ALT and AST serum levels (Figure 4C). Additionally, *Rgs16*‐HTG mice presented significantly larger necrotic areas than *Rgs16*‐NTG mice did (Figure 4D). Furthermore, compared with *Rgs16*‐NTG mice, *Rgs16*‐HTG mice presented higher protein expression levels of BAX and C‐Caspase3 and lower BCL‐2 expression levels upon HIRI, indicating aggravated hepatic apoptosis (Figure 4E). TUNEL staining further confirmed increased apoptosis in *Rgs16*‐HTG mice following HIRI (Figure 4F). The inflammatory response was also intensified in *Rgs16*‐HTG mice, as evidenced by elevated serum cytokine levels and mRNA levels of TNFα, IL‐6, IL‐1β, and CCL2 (Figure 4G,H). Notably, the NF‐κB signaling pathway was activated following RGS16 overexpression (Figure 4I). These findings clearly demonstrate that the hepatic overexpression of RGS16 worsens I/R‐induced liver injury, enhancing both inflammation and apoptosis.

### 2.5 RGS16 Upregulates CXCL1 Expression in a YTHDF3‐Dependent Manner

**FIGURE 5:**
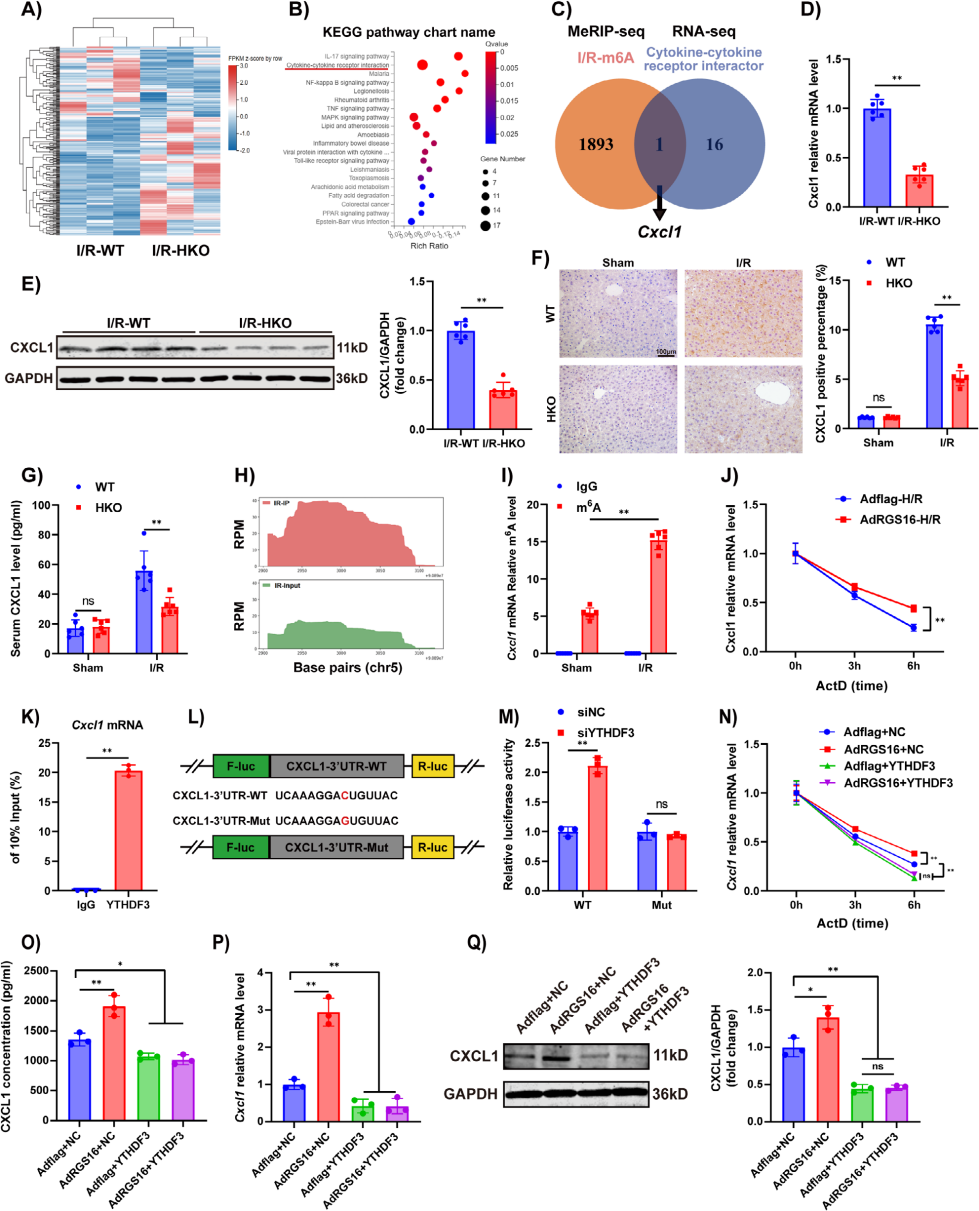
RGS16 upregulates CXCL1 expression in a YTHDF3‐dependent manner. (A–H) In vivo and sequencing‐based analyses. (A) RNA‐seq analysis was performed using liver tissues from WT and *Rgs16*‐HKO mice after HIRI. The heatmap shows DEGs between the two groups, with red indicating upregulated genes and blue indicating downregulated genes. (B) KEGG analysis was performed based on the RNA‐seq data. (C) Venn diagram showing the combined analysis of RNA‐seq and MeRIP‐seq data. (D) Relative mRNA expression levels of CXCL1 in liver tissues from WT and *Rgs16*‐HKO mice after HIRI (*n* = 6). (E) Protein levels of CXCL1 in liver tissues from WT and *Rgs16*‐HKO mice after HIRI (*n* = 6). (F) Immunohistochemical staining of CXCL1 in liver sections from WT and *Rgs16*‐HKO mice after HIRI (*n* = 6). Scale bar = 100 µm. (G) Serum CXCL1 levels in WT and *Rgs16*‐HKO mice after HIRI (*n* = 6). (H) Peak distribution of *Cxcl1* mRNA m^6^A modification derived from the MeRIP‐seq dataset. (I–Q) In vitro analyses. (I) MeRIP‐qPCR analysis of *Cxcl1* m^6^A modification in AML12 cells (*n* = 6). (J) Relative *Cxcl1* mRNA levels in AML12 cells exposed to ActD after adenovirus‐mediated RGS16 overexpression (*n* = 3). (K) RIP‐qPCR analysis of the binding between YTHDF3 and *Cxcl1* mRNA (*n* = 3). (L) Schematic diagram of the CXCL1 mutation site. (M) Luciferase reporter assays were used to verify the binding between YTHDF3 and CXCL1 (*n* = 3). (N) Relative *Cxcl1* mRNA levels in AML12 cells exposed to ActD in the indicated groups (*n* = 3). (O) CXCL1 levels in the conditioned medium were measured by ELISA (*n* = 3). (P, Q) mRNA and protein expression levels of CXCL1 in AML12 cells in the indicated groups (*n* = 3). Data were normalized to GAPDH where applicable and are presented as mean ± SD. Two‐tailed unpaired Student's *t*‐test was used for comparisons between two groups. One‐way ANOVA followed by Tukey's multiple comparison test was used for comparisons among multiple groups. Two‐way ANOVA followed by Tukey's multiple comparison test was used for ActD time‐course experiments. *p < 0.05, **p < 0.01, n.s., not significant.

To investigate how RGS16 promotes inflammation and apoptosis during HIRI, we constructed HIRI models using WT mice and *Rgs16*‐HKO mice and performed RNA sequencing (RNA‐seq) using liver tissues (Figure 5A). The cytokine‒cytokine receptor interaction signaling pathway was subsequently identified via Kyoto Encyclopedia of Genes and Genomes (KEGG) analysis of the differentially expressed genes (DEGs) (Figure 5B). Given the importance of m^6^A modification in posttranscriptional regulation [15], we integrated methylated RNA immunoprecipitation sequencing (MeRIP‐seq) data from our previous study [16] with the RNA‐seq data, identifying *Cxcl1* as a key target (Figure 5C). RGS16 knockdown significantly reduced both mRNA and protein levels of CXCL1 (Figure 5D–G). MeRIP‐seq analysis revealed increased m^6^A modification on *Cxcl1* mRNA after HIRI, confirmed by MeRIP‐qPCR (Figure 5H,I). To further investigate the mechanism, we overexpressed RGS16 in AML12 cells and subjected them to H/R. The half‐life of *Cxcl1* mRNA was significantly prolonged in RGS16‐overexpressing cells compared to controls (Figure 5J). Since m^6^A modification regulates mRNA stability, we hypothesized that a specific reader protein binds to *Cxcl1* mRNA and exerts its effect [17]. Immunoprecipitation and mass spectrometry identified YTHDF3 as a binding partner of RGS16, and RNA immunoprecipitation (RIP) confirmed YTHDF3 binding to *Cxcl1* mRNA (Figure 5K). Using the SRAMP database (http://www.cuilab.cn/sramp), we predicted and mutated the YTHDF3 binding site on *Cxcl1* mRNA (Figure 5L). We first constructed and validated a YTHDF3 siRNA (Figure S2A,B). A subsequent luciferase reporter assay revealed no change in activity after mutation, confirming the specificity of the predicted binding site (Figure 5M). To investigate whether RGS16 regulates *Cxcl1* expression in a YTHDF3‐dependent manner, we generated and verified a YTHDF3 overexpression plasmid (Figure S2C,D). In vitro, YTHDF3 overexpression reversed the RGS16‐induced prolongation of *Cxcl1* mRNA half‐life (Figure 5N). Moreover, overexpressed YTHDF3 further attenuated the RGS16‐mediated increase in both *Cxcl1* mRNA and protein levels (Figure 5O–Q). We next performed an in vivo rescue experiment (Figure S3A). A YTHDF3 overexpression adenovirus was constructed and administered to *Rgs16*‐HTG mice via tail vein injection (Figure S3B,C). Following HIRI, we observed results similar to those obtained in vitro; specifically, the RGS16 overexpression–induced elevation of CXCL1 expression was reversed by YTHDF3 overexpression (Figure S3D,E). Collectively, these findings demonstrate that RGS16 stabilizes *Cxcl1* mRNA and enhances its expression through a YTHDF3‐dependent mechanism.

### 2.6 RGS16 Competitively Inhibits the Binding of PAN3 to the YTHDF3 Protein

**FIGURE 6:**
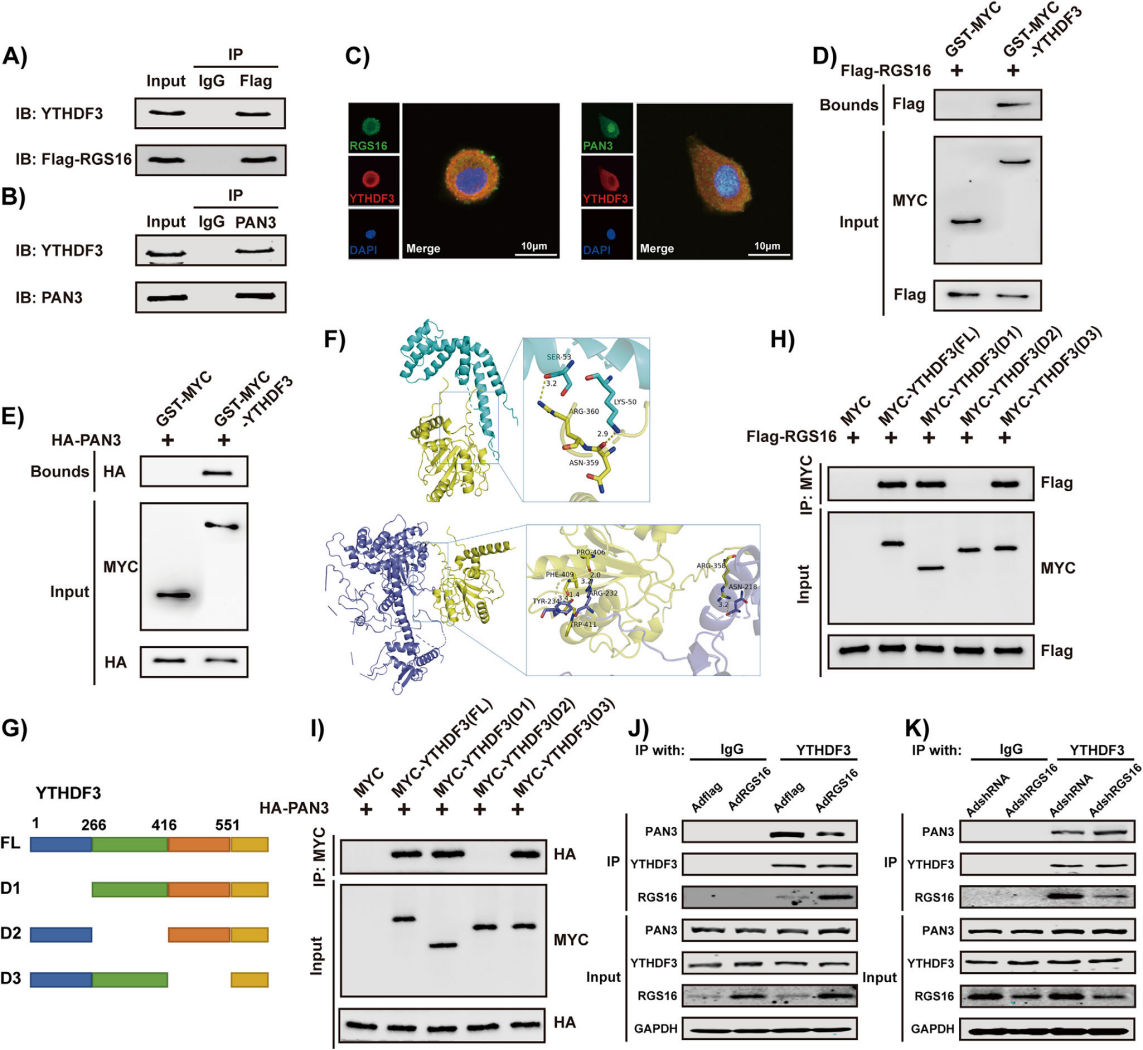
RGS16 competitively inhibits the binding of PAN3 to the YTHDF3 protein. (A) Overexpression of 3×Flag‐RGS16 in AML12 cells. Co‐IP assays were used to identify the interaction between RGS16 and YTHDF3 (*n* = 3). (B) Co‐IP assays were used to identify the interaction between PAN3 and YTHDF3 (*n* = 3). (C) Confocal microscopy was used to identify the colocalization of RGS16 and PAN3 with YTHDF3 (*n* = 3). Scale bars = 10 µm. (D, E) GST‐YTHDF3 protein was purified in vitro, and GST pull‐down assays were used to assess the interaction of RGS16 and PAN3 with YTHDF3 (*n* = 3). (F) Molecular docking analysis of RGS16 and PAN3 binding to the low‐complexity domain of YTHDF3. (G) Schematic diagram showing full‐length YTHDF3 and truncated YTHDF3 fragments D1, D2, and D3. (H, I) Co‐IP assays were performed to determine the specific YTHDF3 domains responsible for binding to RGS16 and PAN3 (*n* = 3). (J, K) Co‐IP assays were used to assess the interaction between PAN3 and YTHDF3 after RGS16 overexpression or knockdown (*n* = 3). Representative images are shown from three independent experiments.

To elucidate the molecular mechanism by which RGS16 regulates CXCL1 expression, we first demonstrated that RGS16 binds to YTHDF3 in AML12 cells via a co‐immunoprecipitation (co‐IP) assay (Figure 6A). YTHDF3 reportedly interacts with PAN3 and recruits PAN2‐PAN3 complex to target mRNA for deadenylation [18]. Therefore, we verified the binding of PAN3 to YTHDF3 in AML12 cells (Figure 6B). Using laser confocal microscopy, we assessed the intracellular distribution of RGS16, YTHDF3, and PAN3 and found that both RGS16 and PAN3 colocalized with YTHDF3 in the cytoplasmic region (Figure 6C). Furthermore, a GST pulldown assay confirmed that the purified GST‐YTHDF3 protein directly bound to RGS16 and PAN3 (Figure 6D,E). Using a molecular docking technique, we subsequently found that both RGS16 and PAN3 bind the low‐complexity structural domain of YTHDF3 (Figure 6F and Figure S4). To validate that finding, we designed different truncations for the protein structural domain of YTHDF3 (Figure 6G). We found that both RGS16 and PAN3 bound to the low‐complexity domain of YTHDF3, as shown by a co‐IP assay (Figure 6H,I). Moreover, the overexpression or knockdown of RGS16 affected the degree of binding between PAN3 and YTHDF3 (Figure 6J,K). These findings suggest that RGS16 competitively inhibits the binding of PAN3 to the YTHDF3 protein, thus influencing the regulation of CXCL1 expression.

### 2.7 The CXCL1‐CXCR2 Axis Mediates RGS16‐Induced Hepatocyte Inflammation and Apoptosis During H/R In Vitro

To elucidate how RGS16 regulates hepatocyte apoptosis and inflammation, we focused on C‐X‐C motif chemokine receptor 2 (CXCR2), which is implicated in liver recovery during HIRI [19]. Since CXCL1 is a major ligand for CXCR2, we hypothesized that RGS16‐CXCL1 signaling exerts an autocrine effect to increase hepatocyte apoptosis and inflammation after H/R. To test this, hepatocytes were pretreated with SB225002, a CXCR2 inhibitor, for 24 h before H/R stimulation. Compared with vehicle treatment, inhibitor treatment significantly reduced LDH levels, increased cell viability, and alleviated the levels of inflammation and apoptosis in hepatocytes (Figure S5A–E). To further investigate the role of CXCR2, we designed a CXCR2‐specific siRNA. Following successful transfection, hepatocytes were subjected to H/R stimulation (Figure S5F). As expected, compared with the siNC group, the siCXCR2 group presented reduced LDH levels, increased cell viability, and alleviated inflammation and apoptosis in hepatocytes (Figure S5G–K). To confirm RGS16's deleterious effects via CXCL1‐CXCR2 signaling, hepatocytes under H/R conditions were treated with recombinant CXCL1 protein and SB225002. High concentrations of CXCL1 in the culture medium induced significant proinflammatory and proapoptotic effects, which were effectively reversed by CXCR2 inhibition (Figure S5L,M). These findings indicate that CXCL1 activation of CXCR2 drives hepatocyte inflammation and apoptosis following H/R injury. Collectively, CXCL1 contributes to hepatocyte apoptosis and inflammation through an autocrine mechanism under H/R conditions.

### 2.8 Hepatocytic RGS16 Promotes Neutrophil Recruitment and NETosis In Vivo and In Vitro

**FIGURE 7:**
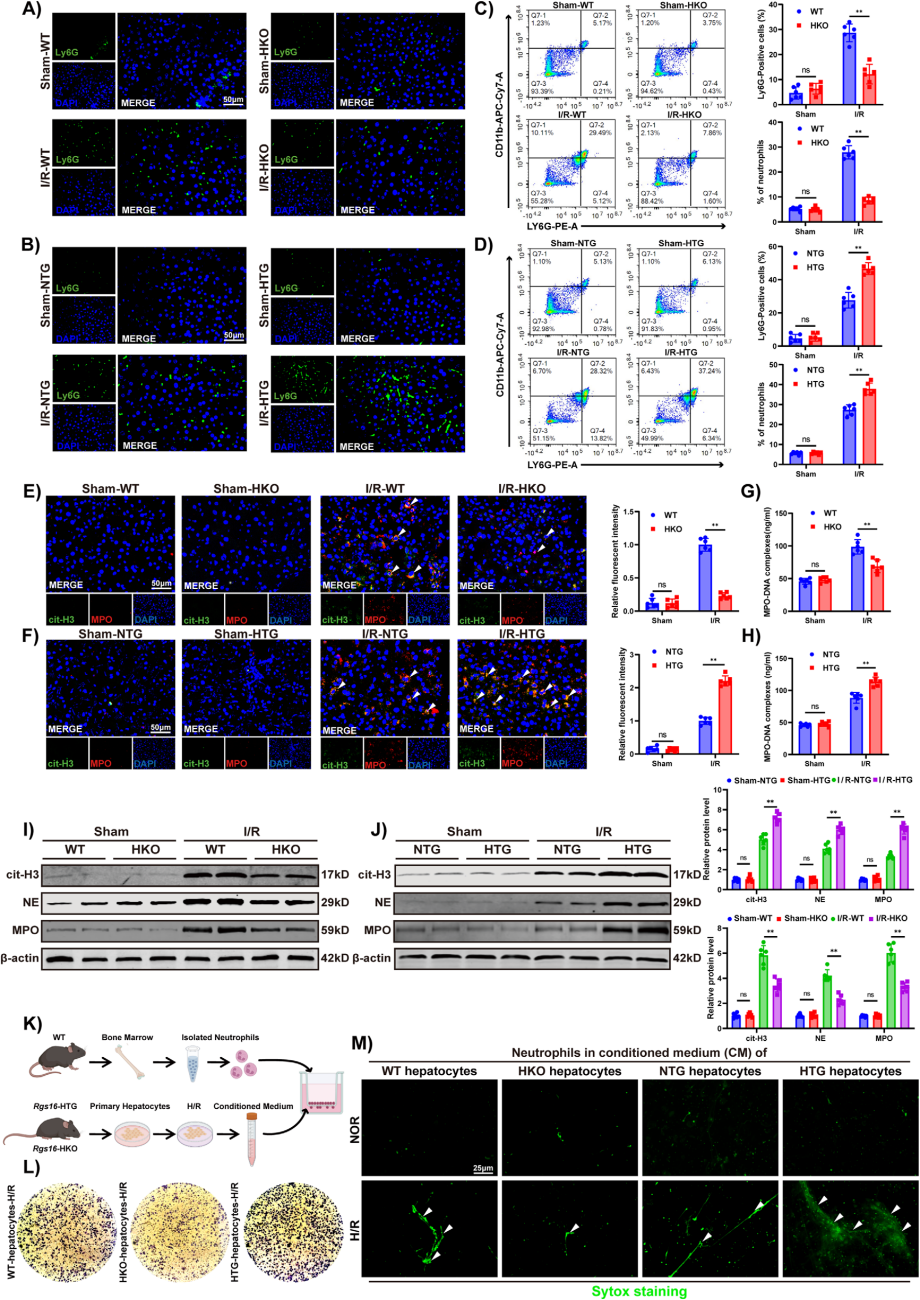
RGS16 promotes the recruitment of neutrophils into the liver and NETosis. (A) Representative immunofluorescence images of Ly6G‐stained liver sections from WT and *Rgs16*‐HKO mice after HIRI (*n* = 6). Scale bar = 50 µm. (B) Representative immunofluorescence images of Ly6G‐stained liver sections from *Rgs16*‐NTG and *Rgs16*‐HTG mice after HIRI (*n* = 6). Scale bar = 50 µm. (C) Flow cytometric quantification of neutrophil infiltration in liver tissues from WT and *Rgs16*‐HKO mice after HIRI (*n* = 6). (D) Flow cytometric quantification of neutrophil infiltration in liver tissues from *Rgs16*‐NTG and *Rgs16*‐HTG mice after HIRI (*n* = 6). (E) Representative immunofluorescence images of cit‐H3 and MPO in liver sections from WT and *Rgs16*‐HKO mice after HIRI (*n* = 6). Scale bar = 50 µm. (F) Representative immunofluorescence images of cit‐H3 and MPO in liver sections from *Rgs16*‐NTG and *Rgs16*‐HTG mice after HIRI (*n* = 6). Scale bar = 50 µm. (G, H) Levels of MPO‐DNA complexes in liver tissues from the indicated groups (*n* = 6). (I, J) Protein levels of NET markers in liver tissues from the indicated groups (*n* = 6). (K) Schematic diagram of bone marrow‐derived neutrophils cultured with conditioned medium from primary hepatocytes isolated from *Rgs16*‐HKO and *Rgs16*‐HTG mice after in vitro H/R challenge (6/6 h). (L) Representative images of crystal violet‐stained neutrophils migrating through the Transwell membrane after stimulation with conditioned medium. (M) Representative images of SYTOX Green staining showing in vitro NET‐related extracellular DNA release (*n* = 3). Scale bar = 25 µm. Data were normalized to GAPDH or β‐actin where applicable and are presented as mean ± SD. Two‐tailed unpaired Student's *t*‐test was used for comparisons between two groups. *p < 0.05, **p < 0.01, n.s., not significant.

CXCL1, a well‐known regulator, regulates neutrophil recruitment and NETosis, exacerbating liver inflammation and apoptosis [20, 21, 22]. Additionally, we observed a positive correlation between elevated RGS16 expression and NETosis in clinical cases following liver surgery (Figure 1G,H), prompting our hypothesis that RGS16 promotes neutrophil recruitment and NETosis in HIRI. To test this, we performed immunofluorescence (IF) staining for Ly6G, a neutrophil‐specific marker, in liver tissues. RGS16 knockout reduced neutrophil accumulation (Figure 7A), while RGS16 overexpression significantly increased neutrophil numbers (Figure 7B). Flow cytometry confirmed these findings, showing a lower percentage of CD11b^+^ Ly6G^+^ neutrophils among CD45^+^ cells in *Rgs16*‐HKO mice compared to WT mice, and a higher percentage in *Rgs16*‐HTG mice (Figure 7C,D), indicating RGS16 promotes neutrophil recruitment. We next assessed NET formation in liver frozen sections. IF staining for cit‐H3 and MPO, two representative NET‐associated markers, showed that Rgs16 deletion reduced NETosis after I/R, whereas Rgs16 overexpression enhanced cit‐H3 and MPO colocalization in the injured liver (Figure 7E,F). Consistently, MPO‐DNA complex ELISAs further confirmed these findings, with *Rgs16*‐HTG mice displaying elevated complex levels in liver tissue, while *Rgs16*‐HKO mice exhibited reduced levels (Figure 7G,H). Western blot analysis revealed that the expression levels of cit‐H3, neutrophil elastase (NE), and MPO were markedly increased in *Rgs16*‐HTG mice and reduced in *Rgs16*‐HKO mice (Figure 7I,J). These in vivo data indicate that hepatocytic RGS16 promotes both neutrophil recruitment and NETosis during HIRI.

In vitro, primary hepatocytes from WT, *Rgs16*‐HKO, and *Rgs16*‐HTG mice were subjected to H/R, and their conditioned medium was used in a Transwell system with WT bone marrow‐derived neutrophils (Figure 7K). After 2 h, the HTG‐H/R group showed significantly more migrating neutrophils than the WT‐H/R group, while the HKO‐H/R group had fewer (Figure 7L). Moreover, SYTOX Green staining showed that conditioned medium from *Rgs16*‐HTG hepatocytes enhanced NET‐related extracellular DNA release, while conditioned medium from *Rgs16*‐HKO hepatocytes attenuated NET formation (Figure 7M). Taken together, these findings suggest that hepatocytic RGS16 enhances the release of pro‐recruitment and pro‐NETosis mediators during HIRI, thereby promoting neutrophil infiltration and NETosis in the injured liver.

### 2.9 RGS16 Drives CXCL1‐Mediated Neutrophil Recruitment and NETosis in HIRI

To further examine whether NET‐associated mechanisms contribute to RGS16‐mediated liver inflammation and apoptosis in vivo, we conducted a phenotypic rescue experiment in mice via the intraperitoneal injection of GSK484, a PAD4 inhibitor, and DNase I. Both GSK484 and DNase I effectively reduced the increase in MPO and cit‐H3 IF levels in *Rgs16*‐HTG mice (Figures S6A and S7A). The protein levels of NETs markers in the liver also confirmed that NETosis was significantly inhibited (Figures S6B and S7B). Additionally, the elevated levels of MPO‐DNA complexes in the livers of *Rgs16*‐HTG mice after HIRI were significantly reduced following GSK484 and DNase I treatment (Figures S6C and S7C). Compared with vehicle treatment, inhibitor treatment alleviated the proinflammatory and proapoptotic effects induced by RGS16 overexpression (Figures S6D,E and S7D,E).

**FIGURE 8:**
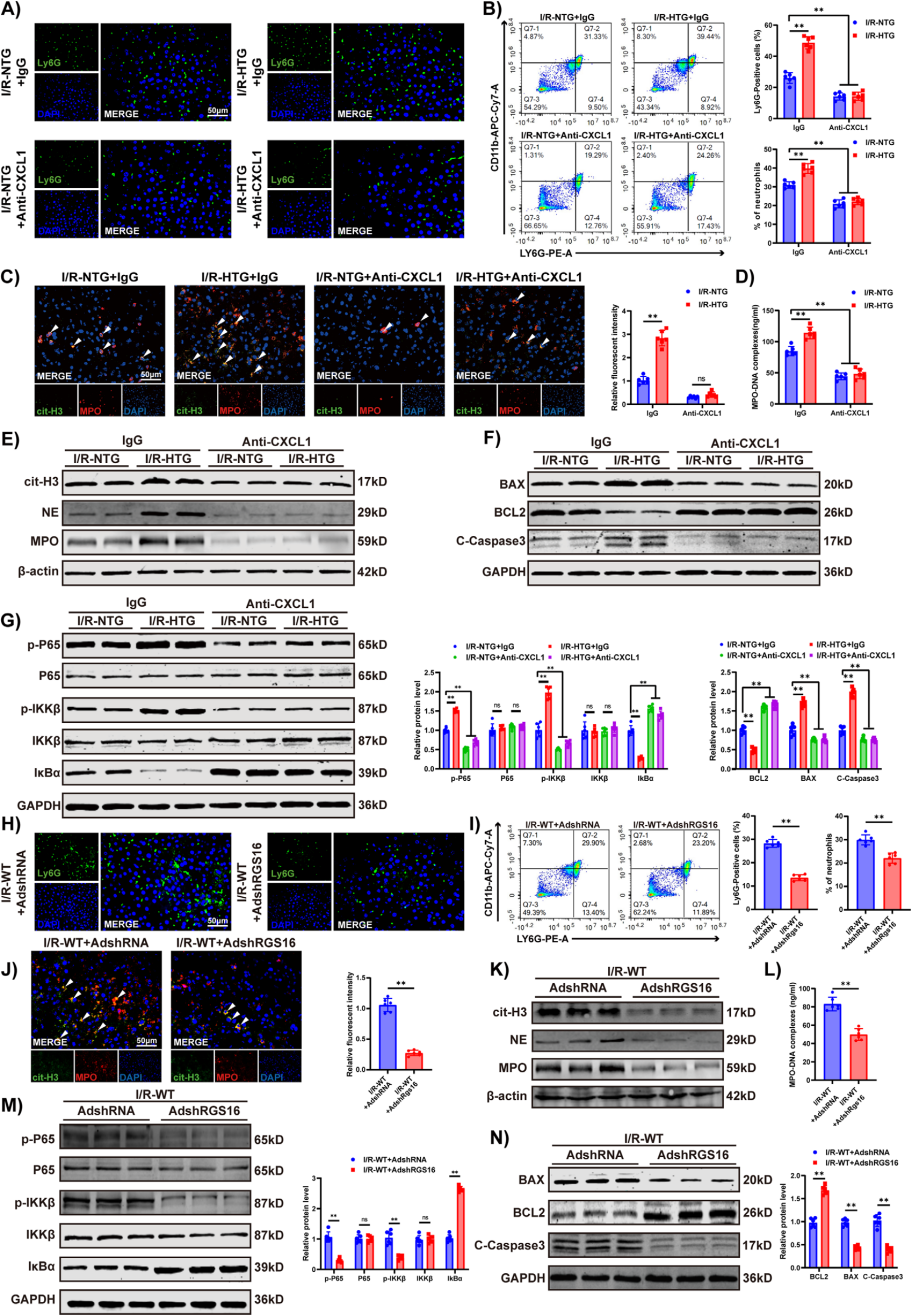
RGS16 promotes neutrophil recruitment to the liver in a CXCL1‐dependent manner and exacerbates HIRI. (A) *Rgs16*‐NTG and *Rgs16*‐HTG mice received IgG or anti‐CXCL1‐neutralizing antibody intraperitoneally and were then subjected to I/R. Representative immunofluorescence images of Ly6G in liver tissues from the indicated groups (*n* = 6). Scale bars = 50 µm. (B) Flow cytometric quantification of neutrophil infiltration in liver tissues from the indicated groups (*n* = 6). (C) Representative immunofluorescence images and quantification of cit‐H3 and MPO in liver tissues from the indicated groups (*n* = 6). Scale bars = 50 µm. (D) Levels of MPO‐DNA complexes in liver tissues from the indicated groups (*n* = 6). (E) Protein levels of NET markers in liver tissues from the indicated groups (*n* = 6). (F) Protein levels of BCL2, BAX, and cleaved caspase‐3 in liver tissues from the indicated groups (*n* = 6). (G) Protein levels of NF‐κB signaling pathway molecules in liver tissues from the indicated groups (*n* = 6). (H) WT mice were injected with AdshRNA or AdshRGS16 via the tail vein and then subjected to I/R. Representative immunofluorescence images of Ly6G in liver tissues from the indicated groups (*n* = 6). Scale bars = 50 µm. (I) Flow cytometric quantification of neutrophil infiltration in liver tissues from the indicated groups (*n* = 6). (J) Representative immunofluorescence images and quantification of cit‐H3 and MPO in liver tissues from the indicated groups (n = 6). Scale bars = 50 µm. (K) Protein levels of NET markers in liver tissues from the indicated groups (*n* = 6). (L) Levels of MPO‐DNA complexes in liver tissues from the indicated groups (*n* = 6). (M) Protein levels of NF‐κB signaling pathway molecules in liver tissues from the indicated groups (*n* = 6). (N) Protein levels of BCL2, BAX, and cleaved caspase‐3 in liver tissues from the indicated groups (*n* = 6). Data were normalized to GAPDH or β‐actin where applicable and are presented as mean ± SD. Two‐way ANOVA followed by Tukey's multiple comparison test was used for comparisons among the IgG‐ and anti‐CXCL1‐treated *Rgs16*‐NTG and *Rgs16*‐HTG groups. Two‐tailed unpaired Student's *t*‐test was used for comparisons between AdshRNA‐ and AdshRGS16‐treated groups. *p < 0.05, **p < 0.01, n.s., not significant.

To investigate whether RGS16 promotes neutrophil recruitment to the liver in a CXCL1‐dependent manner, we intraperitoneally injected anti‐CXCL1 neutralizing antibodies into *Rgs16*‐HTG mice. Ly6G IF staining and flow cytometry confirmed that CXCL1 neutralization effectively inhibited the recruitment of neutrophils to the liver induced by RGS16 overexpression (Figure 8A,B). Accompanying the reduction in infiltrating neutrophils following CXCL1 neutralization, there was a marked decrease in NETosis in the liver (Figure 8C–E and Figure S8). Furthermore, compared with those in the control group, both the cell apoptosis and inflammation associated with RGS16‐induced NET‐related responses in vivo were inhibited by the anti‐CXCL1 neutralizing antibody (Figure 8F,G and Figure S9A–F). These findings indicate that RGS16 promotes neutrophil recruitment and NET‐associated mechanisms in a CXCL1‐dependent manner, thereby contributing to HIRI.

### 2.10 RGS16 Knockdown Attenuates HIRI In Vivo

Finally, to obtain evidence that the suppression of RGS16 is protective against HIRI, we constructed a purified *Rgs16*‐knockdown adenovirus vector. We modulated *Rgs16* expression via tail vein injection in WT mice and then established a HIRI model 72 h post‐injection. The results revealed that RGS16 knockdown via the use of a purified adenovirus significantly reduced neutrophil recruitment and NETosis (Figure 8H–L and Figure S10). The significant inhibition of RGS16 also led to a marked reduction in both the inflammatory response and cell apoptosis (Figure 8M,N and Figure S11A–E). These findings indicate that RGS16 knockdown mitigates inflammation and apoptosis associated with HIRI in vivo, supporting RGS16 as a candidate therapeutic target. The general schematic diagram is shown in Figure S12.

## 3 Discussion

HIRI is a major cause of morbidity and mortality after hepatic resection and transplantation [23]. In this study, we found that RGS16 expression was increased in hepatocytes during HIRI in both in vivo and in vitro models and that RGS16 overexpression exacerbated HIRI, whereas RGS16 knockdown or knockout had the opposite effect. Mechanistically, RGS16 inhibits the degradation of m^6^A‐modified CXCL1 by YTHDF3 through the competitive binding of YTHDF3 to PAN3, and the increased expression of CXCL1 exacerbates the inflammatory response, apoptosis, and NETosis in liver tissues. Importantly, the clinical relevance of RGS16 in HIRI was confirmed in patients undergoing hepatectomy, highlighting its potential as a therapeutic target for HIRI. Notably, the present study mainly focused on the acute injury phase of HIRI. In our time‐course analysis, RGS16 expression peaked at 6 h after HIRI and declined thereafter, suggesting that its role is more closely related to the early injury response. Therefore, whether RGS16 also affects later inflammation resolution, tissue repair, or liver regeneration was not systematically addressed in the current study and warrants further investigation.

The inflammatory response and apoptosis are central to HIRI pathogenesis, and their inhibition can mitigate liver injury [24]. Members of the RGS protein family, including RGS1, RGS5, and RGS6, have been implicated in regulating inflammation and apoptosis in liver diseases [25, 26, 27]. RGS16, another member of the RGS family, is an important regulator of inflammatory responses and modulates immune cell function [28, 29]. In fact, the role of RGS16 in innate immunity has been increasingly recognized. In monocyte‐derived dendritic cells, TLR3 or TLR4 signaling can induce RGS16 expression [30]; moreover, Tian et al. demonstrated that adoptive transfer of macrophages overexpressing RGS16 into mice aggravated HBeAg‐induced liver injury [31]. However, although HIRI is primarily driven by innate immunity, the role of RGS16 in this context has not been previously explored. In this study, we first demonstrated that RGS16 exacerbates sterile inflammation and hepatocyte apoptosis in HIRI. In summary, the interactions among RGS16, YTHDF3, and PAN3 lead to overexpressed CXCL1, increased neutrophil recruitment to the liver, and enhanced NETosis, highlighting the critical role of immune microenvironment modulation in the pathogenesis of HIRI.

CXCL1, a neutrophil chemoattractant, is upregulated in various IRI models [32, 33, 34]. Our study revealed that RGS16 promotes CXCL1 expression during HIRI. Interestingly, our previous m^6^A sequencing data revealed that CXCL1 undergoes m^6^A modification during HIRI. We further demonstrated that RGS16 overexpression increased the half‐life of *Cxcl1* mRNA. This led us to investigate whether RGS16 alters CXCL1 expression by interacting with an m^6^A‐modified protein. Therefore, we overexpressed RGS16 in hepatocytes and used immunoprecipitation combined with mass spectrometry to identify interacting proteins. We identified YTHDF3, an m^6^A reader protein, as a direct binding partner of RGS16.

YTHDF3, one of the well‐studied m^6^A reader proteins, interacts with PAN3 and recruits the PAN2‐PAN3 complex to deadenylate mRNAs, promoting the degradation of somatic cell‐associated genes [18, 35]. To investigate whether RGS16 regulates CXCL1 by modulating the YTHDF3‐PAN3 interaction, we performed molecular docking, revealing that both RGS16 and PAN3 bind to the low‐complexity domain of YTHDF3. Immunoprecipitation assays with truncated YTHDF3 domains confirmed this binding. Additionally, RGS16 overexpression or knockdown altered YTHDF3‐PAN3 binding affinity, indicating that RGS16 competitively inhibits their interaction, thereby stabilizing CXCL1 mRNA by preventing its deadenylation. These results provide mechanistic insights into how RGS16 regulates CXCL1 expression.

Given the secretion properties of CXCL1, we hypothesized that RGS16 enhances CXCL1 release from hepatocytes. Our study demonstrated that RGS16 increases CXCL1 secretion, which exerts autocrine effects by binding to CXCR2 on the hepatocyte surface. This interaction triggers downstream signaling pathways, driving proinflammatory and proapoptotic responses within hepatocytes. CXCR2 activation has been previously linked to amplified inflammation and apoptosis in IRI contexts [36, 37]. Our findings align with those observations, suggesting that RGS16‐mediated CXCL1/CXCR2 signaling represents a critical mechanism in the pathogenesis of HIRI; this pathway not only exacerbates local inflammation but also establishes a proinflammatory microenvironment that may amplify immune‐mediated damage.

Neutrophils, key players in innate immunity, contribute to HIRI through phagocytosis, cytokine release, and NETosis [38, 39]. NETs play a key role in aseptic inflammation during HIRI, exacerbating the inflammatory response and disrupting hepatocyte function [40]. Previous studies have demonstrated that inhibiting NETosis attenuates HIRI in mouse models [41]. In this study, RGS16 overexpression enhanced neutrophil infiltration and NETosis in the liver, suggesting that RGS16‐mediated neutrophil recruitment and NETosis drive HIRI progression. CXCL1, a well‐established neutrophil chemoattractant, was shown to play a central role in this process. Neutralizing CXCL1 effectively reversed the RGS16‐induced inflammation, apoptosis, neutrophil recruitment, and NETosis. These findings highlight the pivotal role of the RGS16‒CXCL1 axis in coordinating neutrophil‐mediated inflammation and apoptosis during HIRI. While prior studies have linked CXCL1‐mediated neutrophil recruitment to tissue injury in other inflammatory contexts [42, 43], our study extends this understanding by connecting RGS16 to CXCL1 regulation and its downstream effects on NETosis, a major contributor to HIRI. It should also be noted that CXCL1 is a pleiotropic chemokine whose functions are not limited to neutrophil recruitment alone. Although our current data most directly support a role for the RGS16–CXCL1 axis in neutrophil/NET‐related injury during the early phase of HIRI, we cannot exclude that sustained CXCL1 elevation may also influence other myeloid populations, including monocytes or macrophages, particularly during later phases of inflammation resolution and tissue repair. This aspect was not systematically investigated in the present study and should be addressed in future work.

Our study underscores the clinical relevance of targeting RGS16 or its downstream effector CXCL1 as a novel therapeutic approach for treating HIRI. Given that RGS16 plays a central role in modulating inflammation and apoptosis in HIRI, inhibiting RGS16 activity may help attenuate liver damage in patients undergoing liver operations. Additionally, targeting CXCL1 could provide an alternative strategy to block neutrophil‐mediated injury and NETosis, both of which are critical drivers of HIRI progression. Our findings suggest that either directly inhibiting RGS16 or disrupting CXCL1/CXCR2 signaling could offer therapeutic benefits by reducing inflammation, apoptosis, and immune cell infiltration in the liver. Clinical trials evaluating small‐molecule inhibitors or biologics that target RGS16 or the CXCL1/CXCR2 axis may therefore hold promise for improving outcomes in patients with HIRI, offering a more effective treatment strategy for this challenging condition.

In conclusion, the dual role of RGS16 in both hepatocyte‐intrinsic signaling and neutrophil‐mediated injury positions it as a central node in the inflammatory cascade that amplifies HIRI. By simultaneously promoting hepatocyte injury and immune cell infiltration, RGS16 drives a feedforward loop of tissue damage, making it an attractive therapeutic target. These findings suggest that targeting RGS16 or its downstream effectors, such as CXCL1, may provide a novel therapeutic approach for mitigating HIRI and improving outcomes after liver transplantation and hepatectomy.

## 4 Materials and Methods

### 4.1 Human Liver Samples

Human liver samples were collected from patients diagnosed with benign liver diseases who underwent partial hepatectomy (*n* = 60) in accordance with the ethical principles of the Declaration of Helsinki. Liver biopsy samples were taken intraoperatively before hepatic resection, specifically prior to portal vein clamping. Additional biopsies were collected after reperfusion and before abdominal closure. In this cohort, the number of vascular occlusions ranged from 1 to 4, and the duration of each occlusion was 15 min. Following collection, the samples were promptly frozen in liquid nitrogen and subsequently stored at −80°C. ALT and AST levels were assessed on the initial POD1 to assess the extent of liver damage. The baseline clinical and operative characteristics of the patients, including age, sex, diagnosis, preoperative laboratory parameters, ischemia method, number of vascular occlusions, intraoperative blood loss, and operation time, are summarized in Table S1. Informed consent forms were signed by all participants.

### 4.2 Animals

The C57BL/6J male mice, aged 6–8 weeks and weighing 18–23 g, were kept in the SPF Laboratory Animal Center of the First Affiliated Hospital of Harbin Medical University under a 12 h light‒12 h dark cycle with free access to food and water. All animal experiments were conducted in compliance with the ARRIVE guidelines and the Guide for the Care and Use of Laboratory Animals. The study protocol was approved by the Ethics Committee of the First Affiliated Hospital of Harbin Medical University (2024139). All efforts were made to minimize animal suffering and to use the minimum number of animals necessary to achieve statistical significance.

### 4.3 Statistical Analysis

All statistical analyses were performed using GraphPad Prism 5.0 (GraphPad Software, San Diego, CA, USA). Data are presented as mean ± SD. The sample size, n, represents independent biological replicates unless otherwise stated. Technical replicates were averaged before statistical analysis. Data were analyzed using two‐tailed unpaired Student's *t*‐test for comparisons between two groups and one‐way ANOVA followed by Tukey's multiple comparison test for comparisons among multiple groups. For paired samples, a two‐tailed paired Student's t‐test was used. For nonparametric data, the Mann–Whitney U test was used. Correlations were analyzed using Pearson's correlation coefficient. *P* < 0.05 was considered statistically significant.

## Author Contributions

This article was equally contributed by X.L., Z.M., and H.Y. X.L. and Z.M. collaborated on designing the research experiments and participated in the process of writing the articles. H.Y., Y.H., Z.L., B.Y., and B.Q. participated in the creation and examination of information. Analysis was also supported by Z.L., Y.Z., Z.F., S.L., S.K., M.B., Y.F., W.T., and N.B. Y.M. initiated the study, organized, designed, and wrote the paper. The final manuscript was read and approved by all authors.

## Funding

The Research Fund of the National Natural Science Foundation of China (82525068, 82370643, YM). Chen Xiaoping Foundation for the Development of Science and Technology of Hubei Province (CXPJJH11900001‐2019349, YM). Scientific Foundation of the First Affiliated Hospital of Harbin Medical University (HYD2020JQ0007, YM). The Key Research and Development Program of Heilongjiang Province (2024ZX12C28, YM).

## Ethics Statement

All animal studies were conducted in strict compliance with the guidelines and regulations of the Committee on the Use of Live Animals in Teaching and Research at Harbin Medical University (2024139). The informed consent was obtained from all participants included in the study, in agreement with institutional guidelines.

## Conflicts of Interest

Authors declare that they have no conflicts of interest.

## Supporting information




**Supporting File**: advs76817‐sup‐0001‐SuppMat.docx.

## Data Availability

The RNA sequencing data used in the current study is publicly available in the GSA database (https://ngdc.cncb.ac.cn/), with submission numbers CRA030842.
