# Supplementary material for: RGS16 Aggravates Hepatic Ischemia‐Reperfusion Injury via Hepatocyte‐Intrinsic Apoptosis/Inflammation & Neutrophil Recruitment/NETosis
Source: Adv Sci (Weinh). 2026 Jul 28:e76817. Online ahead of print. doi: 10.1002/advs.76817 (PMC13410803; doi:10.1002/advs.76817)
Supplement: Supplementary file 1 — Supporting File: advs76817‐sup‐0001‐SuppMat.docx. [file ADVS-9999-e76817-s001.docx]

**Supplementary Materials and Methods**

**Generation of genetically modified mice**

Hepatocyte-specific *Rgs16* knockout mice (*Rgs16*-HKO) and hepatocyte-specific *Rgs16* transgenic mice (*Rgs16*-HTG) were obtained from Cyagen Biosciences (Guangzhou, China), using CRISPR/Cas9 and targeted insertion techniques. For *Rgs16*-HKO mice, two single guide RNAs (sgRNA1 and sgRNA2) were designed to target regions upstream of exon 2 and downstream of exon 4 of the Rgs16 gene. Homology-directed repair was facilitated using single-stranded oligodeoxynucleotides (ssODNs) carrying loxP sites. The sgRNAs, ssODNs, and Cas9 mRNA were microinjected into C57BL/6J zygotes. PCR and sequencing confirmed successful loxP site integration; primers F1/R1 and F2/R2 were used to detect loxP sites, and primers F3/R3 were used to amplify the floxed region. *Rgs16* flox/flox mice were bred with Albumin-Cre mice (Jackson Laboratory, USA) to generate *Rgs16*-HKO mice.

For *Rgs16*-HTG mice, the targeting vector included a “CAG promoter-loxP-PGK-Neo-6*SV40 pA-loxP-Kozak-Rgs16 CDS-rBG pA” cassette, which was cloned and inserted into intron 1 of the ROSA26 locus on chromosome 6. Homology arms were amplified by PCR using a BAC clone as the template. Cas9 protein and gRNAs were coinjected with the targeting vector into fertilized C57BL/6J embryos. Offspring were genotyped by PCR and sequenced to confirm successful knock-in.

Both the *Rgs16*-HKO and *Rgs16*-HTG lines were maintained on a C57BL/6J background. The C57BL/6J wild type (WT) mice used in this study as controls were purchased from Vital River Laboratory Animal Technology (Beijing, China).

**Mouse HIRI model**

The murine warm HIRI model was constructed as described previously^1, 2^. After the mice were anesthetized, midline dissection was conducted to expose the liver. Noninvasive microvascular clips were subsequently used to clamp the left and middle portal vein branches within the liver, thereby interrupting the blood supply. Following a 90-min period of ischemia, the clamps were subsequently released to initiate reperfusion. At designated intervals after reperfusion (3, 6, 12, and 24 h), the mice were anesthetized to obtain liver samples and serum for subsequent analyses. Mice that underwent an identical surgical procedure without occlusion of the vascular system were utilized as sham controls.

For the rescue experiments, the mice were administered an anti-CXCL1 neutralizing antibody (5 mg/kg, R&D Systems) every other day via intraperitoneal injection starting on the seventh day before surgery^3^. DNase I (Cat. 10104159001, 0.1 mg/kg, Roche Diagnostics) was administered daily by intraperitoneal injection for seven days before surgery^4^. Additionally, the mice received daily intraperitoneal injections of the PAD4 inhibitor GSK484 (Cat. GC19184, 4 mg/kg, GLPBIO) starting three days before surgery^5^.

Sample sizes were determined based on prior experience with the HIRI model and pilot experiments, which indicated that the selected numbers were sufficient to detect biologically relevant differences. Mice were randomly assigned to experimental groups using a random number generator, and all procedures were performed in a blinded manner whenever possible. All animals were included in the final analysis unless they exhibited unexpected death unrelated to the experimental procedure (e.g., anesthesia accident). No other exclusion criteria were pre-established.

**Liver function assessment**

Serum ALT and AST levels were quantified using commercially available kits from the Nanjing Jiancheng Institute of Bioengineering (Nanjing, China), following the manufacturer's protocols. This analysis was conducted to evaluate the degree of hepatic injury in the experimental subjects.

**Cell culture and hypoxia/reoxygenation (H/R) model**

HEK293T cells and the AML12 hepatocyte cell line were procured from the Type Culture Collection of the Chinese Academy of Sciences (Beijing, China). Primary hepatocytes were isolated from the liver as previously described^1^, and cells were cultured in DMEM supplemented with 10% fetal bovine serum under a humidified atmosphere with 5% CO2 at 37℃. In order to simulate an HIRI model *in vitro*, cells underwent H/R treatment. AML12 and primary hepatocytes were subjected to hypoxic conditions (1% oxygen) in glucose-free DMEM (Gibco, USA) for 6 h, followed by cultivation under normoxic conditions.

**Histological hematoxylin and eosin (H&E) and immunohistochemical (IHC) staining**

The extent of necrosis in the ischemic lobes was assessed using H&E staining. The results of the assessment were evaluated by quantifying the necrotic area. The necrotic area was calculated as the percentage of the total tissue area using ImageJ software. The expression of RGS16 and CXCL1 was investigated via IHC similar to what we previously reported^6^. The number or percentage of positively stained cells was quantified using ImageJ software by an investigator blinded to the experimental groups.

**Immunofluorescence (IF) and confocal microscopy**

The detailed procedures of IF have been previously described^6^. Confocal microscopy was used to assess the expression of Ly6G, CitH3, and MPO in mouse liver tissue, and the localization of RGS16, YTHDF3, and PAN3 in AML12 cells. In short, cells were cultured in confocal dishes, fixed, permeabilized and blocked. Then, the cells were incubated with primary antibody at 4 °C overnight and then with fluorescent secondary antibody at 37 °C for 1 hour. The nuclei were visualized with DAPI. Images were obtained by confocal microscopy (Olympus, Japan) at room temperature. The primary antibodies used were rabbit anti-Ly6G (Cat. 88876, Cell Signaling Technology), rabbit anti-CitH3 (Cat. ab281584, Abcam), goat anti-MPO (Cat. AF3667, R&D systems), mouse anti-RGS16 (Cat. sc-166083, Santa Cruz Biotechnology), mouse anti-PAN3 (Cat. sc-376434, Santa Cruz Biotechnology) and rabbit anti-YTHDF3 (Cat. 25537-1-AP, Proteintech). The fluorescent secondary antibodies used were Donkey Anti-Rabbit IgG (Cat. BA1146, BOSTER), Donkey Anti-Goat IgG (Cat. P0173, Beyotime), Goat anti-Mouse IgG (Cat. A-11005, Thermo Fisher), Goat anti-Rabbit IgG (Cat. A-11008, Thermo Fisher).

**TUNEL staining**

TUNEL assay (Roche, Switzerland) was performed according to the manufacturer’s instructions to detect apoptosis levels *in vivo* and quantified TUNEL-positive cells in ten randomly selected × 800 high-power fields under microscopy.

**Enzyme-linked immunosorbent assay (ELISA)**

The levels of TNF-α, IL-6, IL-1β, and CCL2 in serum and cell culture medium were measured using ELISA kits (R&D Systems, USA) according to the manufacturer’s instructions. The MPO-DNA contents in human and mouse liver tissues were measured using Human MPO-DNA ELISA Kit (ml342558, mlBio) and Mouse MPO-DNA ELISA Kit (ml338974-J, mlBio), respectively. The Mouse CXCL1 ELISA Kit (RK00038, ABclonal) was used according to the manufacturer’s instructions to detect the concentration of cell-free supernatants and serum of mice.

**Cell viability and apoptosis assay**

Cell viability was assessed by the Cell Counting Kit-8 (Dojindo Molecular Technologies, China) according to the instructions of the manufacturer. Cell cytotoxicity was assessed by measuring lactate dehydrogenase (LDH) release into the medium by necrotic cells using an LDH Cytotoxicity Assay Kit (Promega, USA).

**Plasmid construction**

To construct the expression vectors, we inserted full-length or fragments of coding sequence (CDS) regions into empty vectors. Briefly, full-length and fragmented regions of mouse RGS16 were amplified from mouse complementary DNA (cDNA) and cloned into the pcDNA5-Flag vectors. Full-length mouse YTHDF3 and D1, D2, D3 truncations of YTHDF3 were cloned into the pcDNA5-MYC vector. Full-length mouse PAN3 sequences were individually inserted into the pcDNA5-HA vector. All the plasmid were synthesized by Sangon (Shanghai, China).

**Adenoviral vector construction and infection**

An adenoviral vector-mediated RGS16 overexpression plasmid (AdRGS16-Flag) and three mouse shRGS16 constructs to generate AdshRGS16 adenoviruses were synthesized by HANBIO (Shanghai, China) (**Supplementary Table 2)**. Specifically, the RGS16 coding region lacking the AHD1 structural domain (RGS16-DAHD1) was cloned into the pShuttle-CMV vector under the control of the CMV promoter to generate the AdRGS16DAHD1-Flag adenovirus. The above plasmids were recombined with the pAdEasy backbone vector and then transfected into HEK293 cells using TurboFect transfection reagent (R0531; Thermo Fisher Scientific). Adenoviruses were generated using an AdEasy Adenoviral Vector System Kit (240009, Agilent Technologies, USA). Recombinant adenoviruses were plaque purified and titered to 10^11^ plaque-forming units (PFU) per milliliter. Adflag and AdshRNA (Mature scrambled RNA sequence: CAACAAGATGAAGAGCAGGAA) were used as controls. For *in vivo* experiments, 5 x 10^9^ PFU of adenovirus was injected into mice via tail vein. For *in vitro* experiments, the multiplicity of infection (MOI) of the adenovirus used was 200.

Recombinant adenovirus carrying the mouse Ythdf3 gene (GV315, CMV-MCS-SV40-EGFP) was synthesized by Genechem (Shanghai, China). The adenovirus contained an EGFP reporter and had a titer of 1.0×10^10^ PFU. For *in vivo* overexpression, mice were injected via the tail vein with 1×10^10^ PFU of adenovirus per mouse. Control mice received an equal titer of empty vector adenovirus (GV315-EGFP) under the same conditions.

**Small interfering RNA transfection**

Small interfering RNAs (siRNA) specifically targeting YTHDF3 was synthesized by GenePharma Company (Shanghai, China). siRNA specifically targeting CXCR2 were synthesized by HANBIO Company (Shanghai, China). AMl12 cells or mice primary hepatocytes were transfected by jetPRIME Polyplus kit (France) according to the manufacturer’s instructions. qRT-PCR was performed at 48 h of transfection and immunoblotting analysis was performed after 72 h. All the siRNA sequences are listed in **Supplementary Table 2**.

**RNA isolation and quantitative real-time PCR**

Total RNA from tissues and cells was isolated using an RNA Miniprep Kit (Axygen, China), followed by cDNA synthesis with a Reverse Transcription Kit (Applied Biosystems). Real-time PCR was performed using SYBR Green (Roche, USA) on an ABIPRISM 7500HT instrument (Applied Biosystems, USA). The expression of mRNA was normalized to that of GAPDH. The primer sequences are shown in **Supplementary Table 3**.

**RNA Sequencing (RNA-seq) analysis and Gene enrichment analysis**

Total RNA was extracted from mouse liver tissues using Trizol (Accurate Biology). Then the library construction and RNA-seq were performed using DNBseq technology by the Beijing Genomics Institute. Differential expression analysis was performed using the DESeq2 (v1.4.5) with Q value ≤ 0.05 (or FDR ≤ 0.001). The heatmap was drawn by pheatmap (v1.0.8) according to the gene expression difference in different samples. To take insight to the change of phenotype, GO (http://www.geneontology.org/) and KEGG (https://www.kegg.jp/) enrichment analysis of annotated different expression gene was performed by Phyper based on Hypergeometric test. The significant levels of terms and pathways were corrected by Q value with a rigorous threshold (Q value ≤ 0.05).

**m^6^A RIP-qPCR and RIP-qPCR analysis**

For detection of m^6^A modification level of Cxcl1 mRNA, refer to the instructions of the MeRIP^TM^ m^6^A Transcriptome Profiling Kit (RiboBio, China). In short, 200 μg total RNA was extracted from the cells, and anti-m6A magnetic beads were prepared after fragmentation. The fragmented RNA was incubated with the magnetic beads at 4 ℃ for 2 h. After RNA elution and recovery, reverse transcription and qRT-PCR were performed. RIP analysis was performed using the BersinBio^TM^ RNA Immunoprecipitation (RIP) Kit. Briefly, about 2×10^7 cell sample were collected and treated with a RIP lysate containing a protease inhibitor cocktail and RNase inhibitors. The DNA was removed and equilibrated with proteinA/G beads, then the beads with YTHDF3 or IgG antibodies were collected and incubated at 4 °C overnight. Finally, RNA was purified and assayed for RNA concentration, reverse transcription and subjected to qRT-PCR. Primers for qRT-PCR are listed in **Supplementary Table 4**.

**Molecular docking**

The protein sequences for RGS16, YTHDF3, and PAN3 were all obtained from UniProt(http://www.UniProt.org/). Molecular docking was performed using AutoDock Tools software^7^.

**RNA stability assay**

Cells were cultured in 6-well plates and transfected with adenovirus or siRNA for 48 h. Then Cells were exposed to 5 µg/mL of actinomycin D (APExBIO Technology, USA) or Dimethyl sulfoxide (DMSO) for 3 h and 6 h, respectively. Total RNA extracts were obtained for further qRT-PCR and the degradation rate of Cxcl1, and the mRNA lifetime were calculated.

**Western blot**

RIPA buffer containing protease and phosphatase inhibitors was prepared to lyse liver tissue and cells to extract protein under ice condition. The protein samples were then subjected to electrophoresis in an SDS-PAGE gel and transferred to nitrocellulose membranes. Primary antibodies were prepared according to the instructions and membranes were incubated at 4 °C overnight. The membranes were washed with PBST on the second day and probed with Irdye 800CW secondary antibody (LI-COR, USA). Images were imaged by the Odyssey Imaging System (LI-COR, USA). Information on primary antibodies is provided in **Supplementary Table 5**.

**Co-immunoprecipitation and LC-MS/MS**

Cells were lysed with ice-cold IP lysis buffer (containing protease and phosphatase inhibitors) for 30 min, and the supernatant obtained by centrifugation was immunoprecipitated with Flag or other primary antibodies or negative control IgG at 4 °C on a rotator. The next day, protein complexes were precipitated with A/G plus agarose beads (Santa Cruz, USA) for 4 h at 4 °C. Samples were washed 4-6 times with IP buffer. Subsequently, the beads in loading buffer were boiled in a metal bath for 10 mins to collect the samples. LC-MS/MS analysis was performed by Aksomics (Shanghai, China).

**Glutathione S-transferase (GST) pulldown assays**

GST and GST-YTHDF3 proteins expressed in bacteria were purified by GST tag purification kit (Beyotime, China) according to the manufacturer's protocol. Flag or HA-tagged RGS16 and PAN3 were harvested by corresponding purification kits (Beyotime, China). GST-YTHDF3 fusion protein was added to HEK-293T cells transfected with Flag-tagged RGS16 and HA-tagged PAN3, respectively, and lysis were incubated at 4 °C for 6 h. Bound proteins were then analyzed via western blotting.

**Luciferase reporter assay**

cDNAs containing full-length 3’UTR of Cxcl1 were cloned into pGL3-control vectors (Promega, USA) which was comprised of firefly luciferase(F-luc). YTHDF3 binding sites located at the Cxcl1 gene were mutated from “UCAAAGGA**C**UGUUAC” to “UCAAAGGA**G**UGUUAC”. Pre-treated AML12 cells were first seeded into 12-well plates and then co-transfected with the pRL-TK plasmid (renilla luciferase reporter vector, R-luc) and wild-type or mutant Cxcl1 reporter plasmids using Lipofectamine 3000 for 24-36 h. After harvesting the cells, luciferase activity was detected using the Dual-Glo Luciferase System (Promega, USA).

**Preparation of Single-Cell Suspension from Mouse Liver Tissue**

Fresh mouse liver tissue was collected and stored in tissue preservation solution (Cat. 130-100-008, Miltenyi Biotec) for processing within 24 hours. After washing the tissue with 1× PBS, it was cut into small pieces and ground in sterile 1× PBS to create a single-cell suspension. The suspension was filtered through a 200-mesh filter and resuspended in Percoll solution (Cat. P8370, Solarbio), collecting the cells between the 40% and 70% Percoll layers. After lysing red blood cells with red blood cell lysis buffer, the cells were resuspended in 100 μL of 1× PBS to prepare a single-cell suspension for flow cytometry staining.

**Flow cytometry for hepatic immune cells**

To prepare the flow cytometry staining, dissolve 50 μL of DMSO in LIVE/DEAD™ Fixable Dead Cell Stain (Cat. L34965, Invitrogen) until fully dissolved. Add 0.1 μL of the dead/live dye to the prepared cell suspension and incubate on ice in the dark for 30 minutes. Stain the cells using Brilliant Violet 785™ anti-mouse CD45 (Cat. 103149, BioLegend), PE anti-mouse Ly-6G/Ly-6C (Gr-1) (Cat. 108407, BioLegend), and APC/Cyanine7 anti-mouse/human CD11b (Cat. 101225, BioLegend), following the instructions for dilution. Incubate in the dark at 4°C for 30-60 minutes. After staining, add 1 mL of 1× PBS, centrifuge at 200-300 g for 5 minutes, discard the supernatant, and resuspend the cell pellet in 200 μL of 1× PBS. Finally, filter the suspension through a 200-mesh filter before analyzing with the NovoCyte 3110 (Agilent).

**Neutrophil isolation and NETs detection**

Neutrophils were isolated using the Neutrophil isolation kit (Cat. 130-097-65, Miltenyi Biotec) and following the manufacturer’s protocol. Neutrophils were seeded at 1×10^5^ cells per well in a 24-well plate and incubated in serum-free RPMI 1640 medium for 3 hours. Afterward, PMA and the conditioned medium from primary hepatocytes derived from transgenic mice post-H/R treatment were added. After 2 hours, 200 nM SYTOX Green (Cat. S7020, Thermo Fisher) was introduced, and 15 minutes later, nuclei were stained with DAPI. Images were captured using a fluorescence microscope.

**Chemotaxis assay**

24-well plates with 3μm PET membrane inserts (Cat. TCS019024, Biofil) were coated with 2% BSA solution at 37°C for 1 hour, followed by two washes with PBS. Primary neutrophils (5×10^5^) from WT mice were resuspended in 100 μL HBSS and seeded into the upper chamber. Conditioned medium from H/R-treated primary hepatocytes of transgenic mice (600 μL) was added to the lower chamber. After incubation at 37°C for 2 hours, the cells were fixed with methanol for 5 minutes, followed by staining with 1% crystal violet (Cat. G1071, Solarbio) at room temperature for 5 minutes. Images were captured using an optical microscope (magnification, ×100; Nikon).

**References:**

1. Wang C, Yu H, Lu S, et al. LncRNA Hnf4αos exacerbates liver ischemia/reperfusion injury in mice via Hnf4αos/Hnf4α duplex-mediated PGC1α suppression. Redox Biol. 2022;57:102498.

2. Yu H, Wang C, Qian B, et al. GRINA alleviates hepatic ischemia‒reperfusion injury-induced apoptosis and ER-phagy by enhancing HRD1-mediated ATF6 ubiquitination. J Hepatol. 2025;83(1):131-45.

3. Sellau J, Groneberg M, Fehling H, et al. Androgens predispose males to monocyte-mediated immunopathology by inducing the expression of leukocyte recruitment factor CXCL1. Nature Communications. 2020;11(1):3459.

4. Peer V, Abu Hamad R, Berman S, et al. Renoprotective Effects of DNAse-I Treatment in a Rat Model of Ischemia/Reperfusion-Induced Acute Kidney Injury. Am J Nephrol. 2016;43(3):195-205.

5. Du M, Yang L, Gu J, et al. Inhibition of Peptidyl Arginine Deiminase-4 Prevents Renal Ischemia-Reperfusion-Induced Remote Lung Injury. Mediators Inflamm. 2020;2020:1-14.

6. Lu S, Ke S, Wang C, et al. NNMT promotes the progression of intrahepatic cholangiocarcinoma by regulating aerobic glycolysis via the EGFR-STAT3 axis. Oncogenesis. 2022;11(1).

7. Forli S, Huey R, Pique ME, et al. Computational protein–ligand docking and virtual drug screening with the AutoDock suite. Nat Protoc. 2016;11(5):905-19.

**Supplementary figures**

**
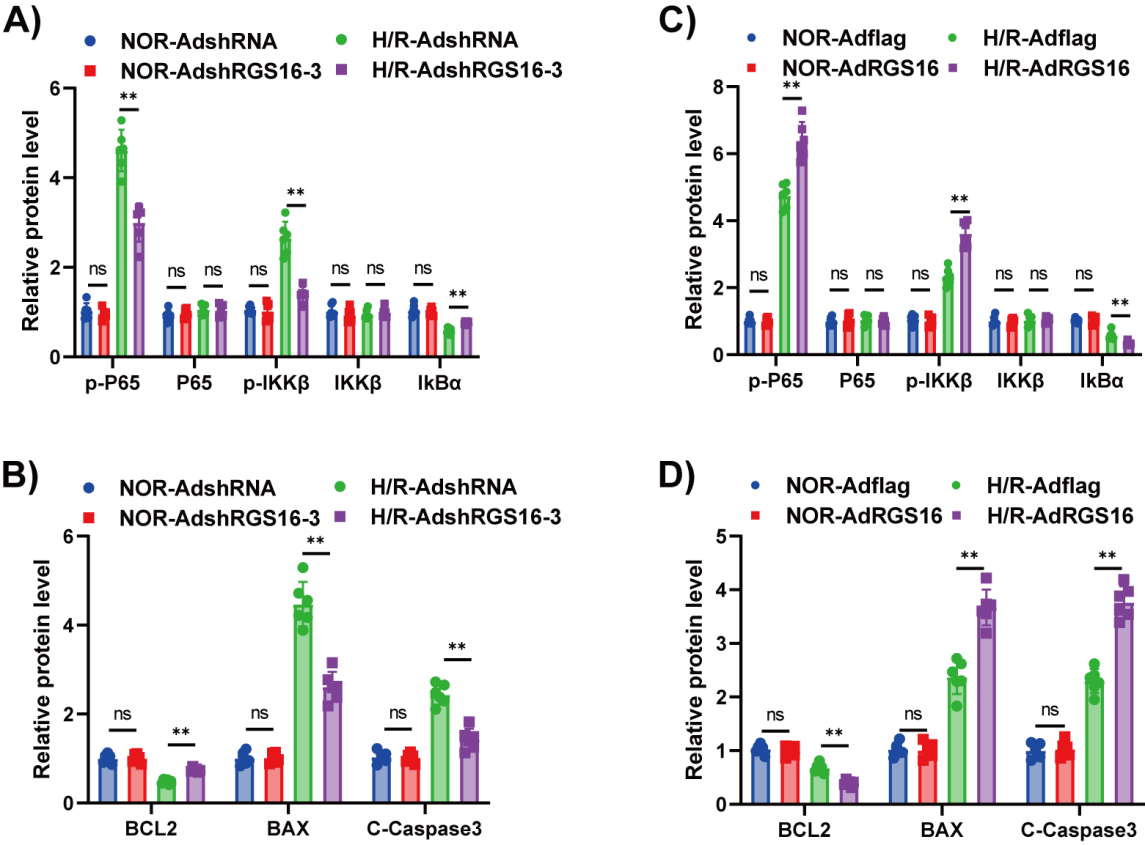
**

**Supplementary Figure 1. Quantification of protein levels from *in vitro* experiments by Western blot.**
(A) Quantification of NF-κB signaling pathway molecules in RGS16-knockdown hepatocytes following hypoxia/reoxygenation (H/R) stimulation (n = 6). (B) Quantification of BCL2, BAX, and cleaved caspase-3 protein levels in RGS16-knockdown hepatocytes following H/R stimulation (n = 6). (C) Quantification of NF-κB signaling pathway molecules in RGS16-overexpressing hepatocytes following H/R stimulation (n = 6). (D) Quantification of BCL2, BAX, and cleaved caspase-3 protein levels in RGS16-overexpressing hepatocytes following H/R stimulation (n = 6). Protein levels were normalized to GAPDH. Data are presented as mean ± SD and are representative of three independent experiments. Two-tailed unpaired Student’s t-test was used for comparisons between two groups, and one-way ANOVA followed by Tukey’s multiple comparison test was used for comparisons among multiple groups. **P < 0.01; n.s., not significant.


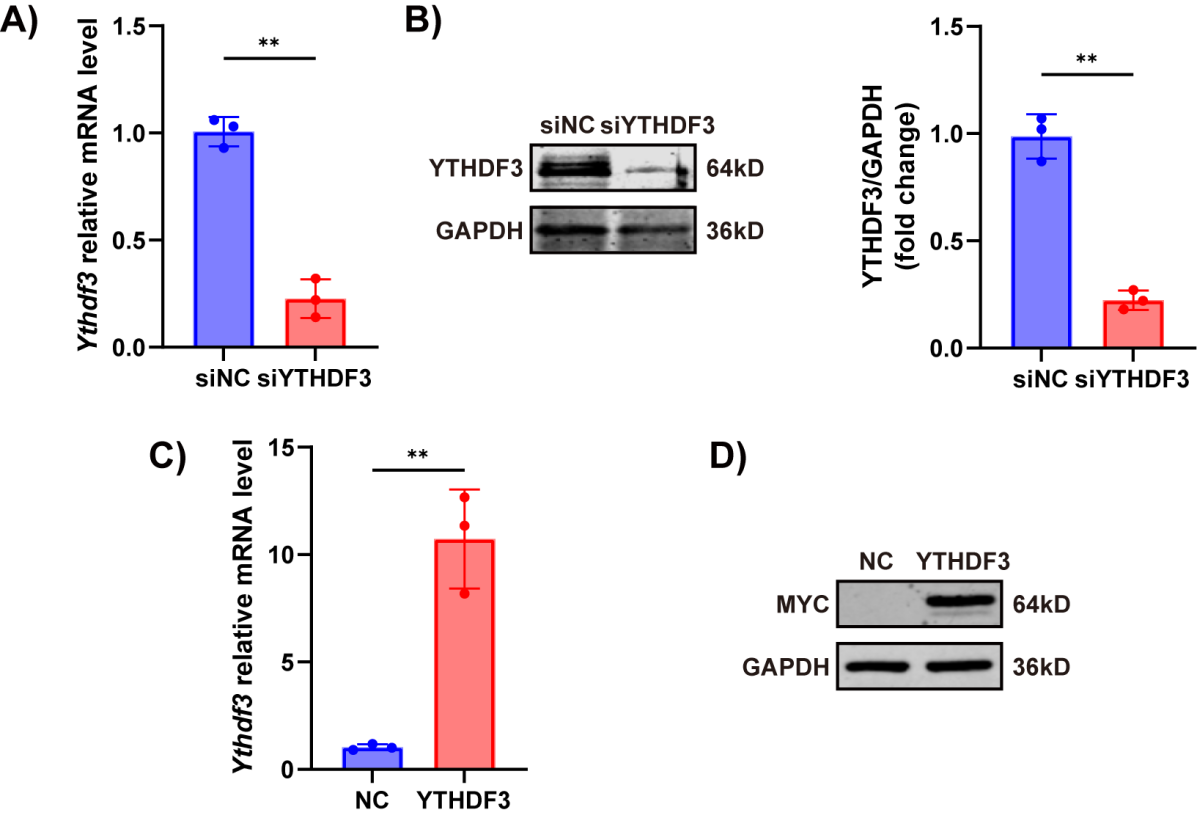


**Supplementary Figure 2. Validation of plasmid transfection efficiency for YTHDF3.**
(A) qRT-PCR analysis of YTHDF3 mRNA levels after transfection with YTHDF3-specific siRNA in cells (n = 3). (B) Western blot analysis of YTHDF3 protein levels after transfection with YTHDF3-specific siRNA in cells (n = 3). (C) qRT-PCR analysis of YTHDF3 mRNA levels after transfection with the YTHDF3-MYC overexpression plasmid in cells (n = 3). (D) Western blot analysis of YTHDF3 protein levels after transfection with the YTHDF3-MYC overexpression plasmid in cells (n = 3). mRNA and protein levels were normalized to GAPDH. Data are presented as mean ± SD and are representative of three independent experiments. Two-tailed unpaired Student’s t-test was used for comparisons between two groups. **P < 0.01.


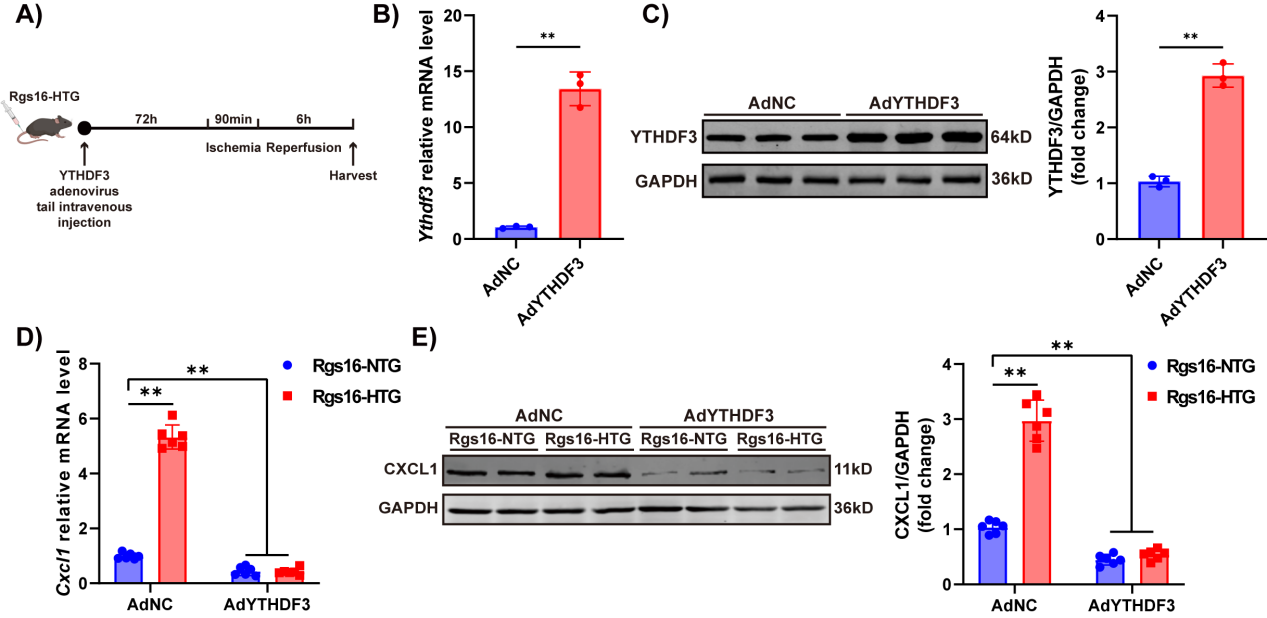


**Supplementary Figure 3. YTHDF3 overexpression reverses the RGS16-induced elevation of CXCL1 expression *in vivo*.**
(A) Schematic diagram of the study design. (B) qRT-PCR analysis of YTHDF3 mRNA levels after tail vein injection of YTHDF3-overexpressing adenovirus in mice (n = 3). (C) Western blot analysis of YTHDF3 protein levels after tail vein injection of YTHDF3-overexpressing adenovirus in mice (n = 3). (D-E) mRNA and protein expression levels of CXCL1 in liver tissues from the indicated groups (n = 6). mRNA and protein levels were normalized to GAPDH. Data are presented as mean ± SD and are representative of three independent experiments. Two-tailed unpaired Student’s t-test was used for comparisons between two groups, and one-way ANOVA followed by Tukey’s multiple comparison test was used for comparisons among multiple groups. **P < 0.01.


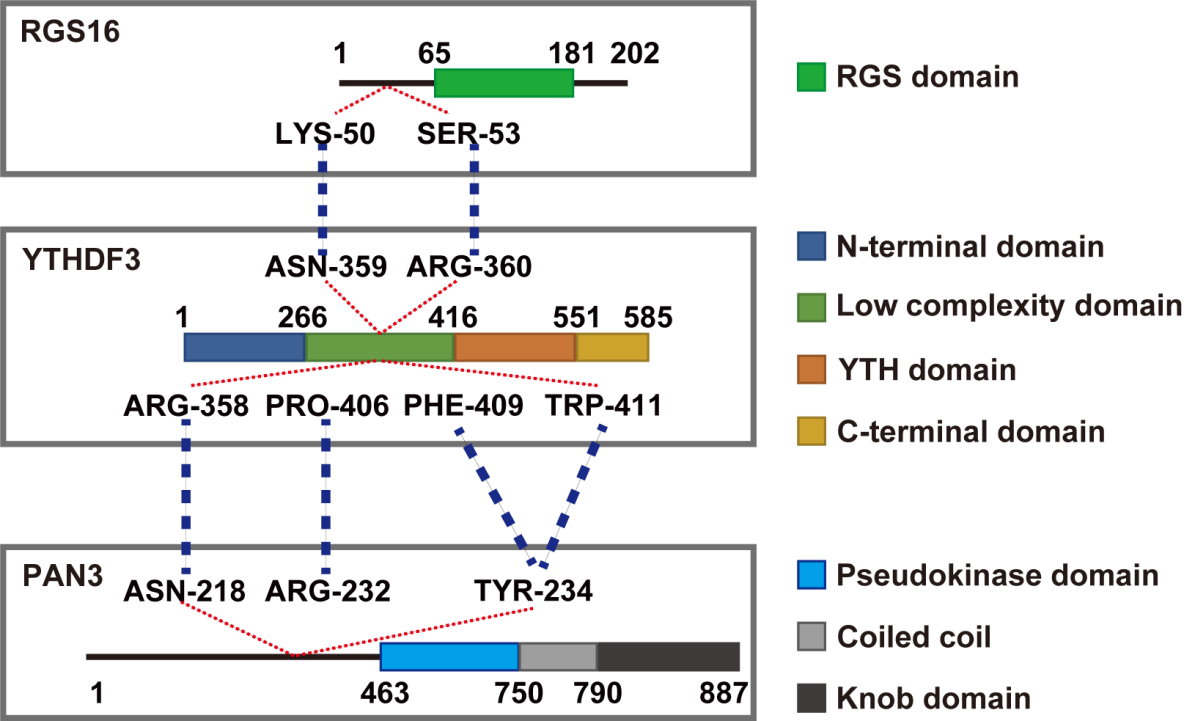


**Supplementary Figure 4. Schematic diagram of the molecular regions of RGS16 and PAN3 that respectively interact with YTHDF3.**


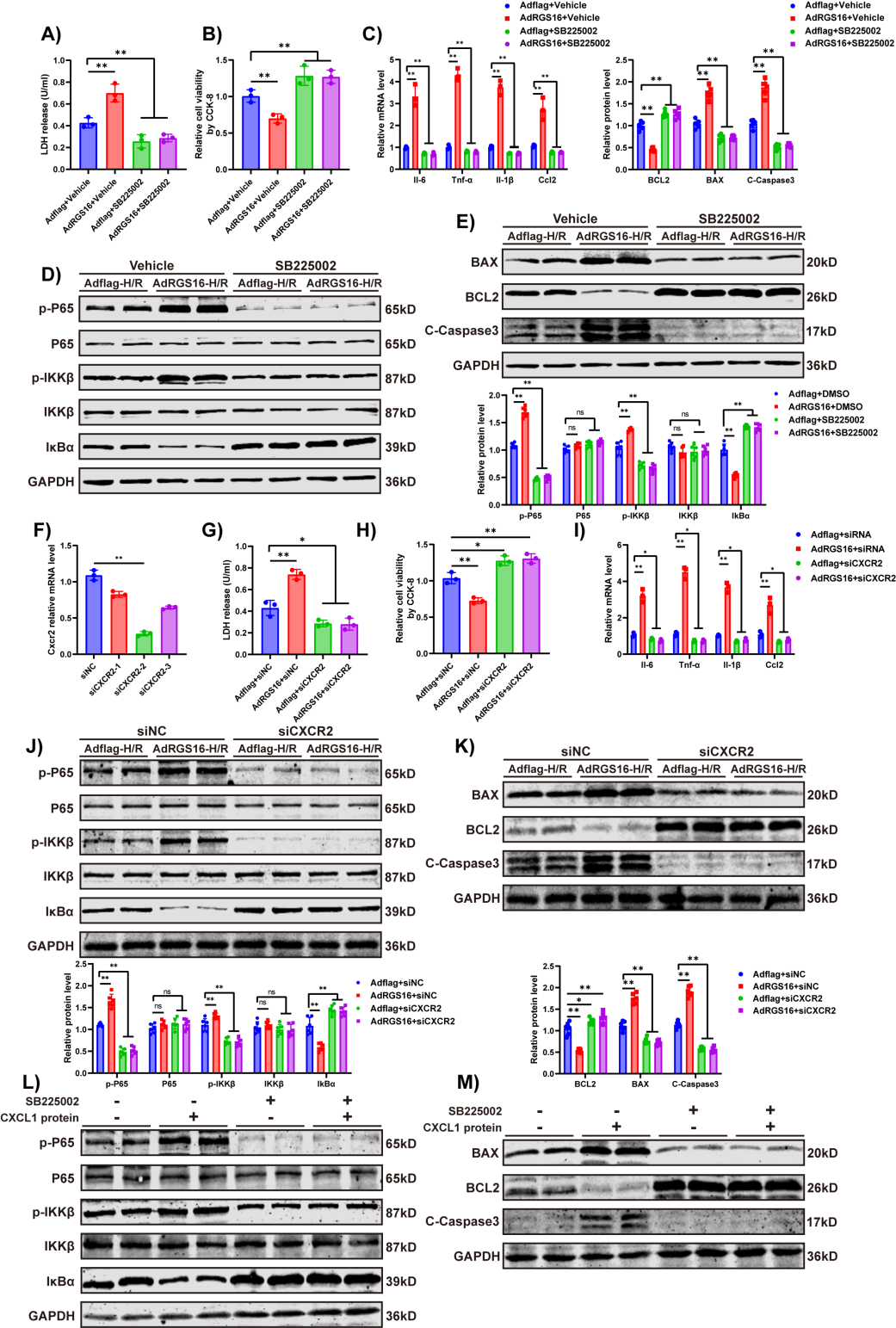


**Supplementary Figure 5. The CXCL1-CXCR2 axis mediates RGS16-induced hepatocyte inflammation and apoptosis during H/R *in vitro*.**

(A) LDH release levels in primary hepatocytes following SB225002 treatment after H/R stimulation (n = 3). (B) CCK-8 assay of primary hepatocytes following SB225002 treatment after H/R stimulation (n = 3). (C) Relative mRNA expression levels of proinflammatory cytokine/chemokine genes in primary hepatocytes following SB225002 treatment after H/R stimulation (n = 3). (D) Protein levels of NF-κB signaling pathway molecules in primary hepatocytes following SB225002 treatment after H/R stimulation (n = 6). (E) Protein levels of BCL2, BAX, and cleaved caspase-3 in primary hepatocytes following SB225002 treatment after H/R stimulation (n = 6). (F) qRT-PCR analysis of Cxcr2 mRNA levels after transfection with CXCR2-specific siRNA in cells (n = 3). (G) LDH release levels in primary hepatocytes following CXCR2 siRNA transfection after H/R stimulation (n = 3). (H) CCK-8 assay of primary hepatocytes following CXCR2 siRNA transfection after H/R stimulation (n = 3). (I) Relative mRNA expression levels of proinflammatory cytokine/chemokine genes in primary hepatocytes following CXCR2 siRNA transfection after H/R stimulation (n = 3). (J) Protein levels of NF-κB signaling pathway molecules in primary hepatocytes following CXCR2 siRNA transfection after H/R stimulation (n = 6). (K) Protein levels of BCL2, BAX, and cleaved caspase-3 in primary hepatocytes following CXCR2 siRNA transfection after H/R stimulation (n = 6). (L) Protein levels of NF-κB signaling pathway molecules in primary hepatocytes following SB225002 treatment with or without recombinant CXCL1 protein after H/R stimulation (n = 6). (M) Protein levels of BCL2, BAX, and cleaved caspase-3 in primary hepatocytes following SB225002 treatment with or without recombinant CXCL1 protein after H/R stimulation (n = 6). mRNA and protein levels were normalized to GAPDH where applicable. Data are presented as mean ± SD and are representative of three independent experiments. Two-tailed unpaired Student’s t-test was used for comparisons between two groups, and one-way ANOVA followed by Tukey’s multiple comparison test was used for comparisons among multiple groups. **P < 0.05, **P < 0.01; n.s., not significant.


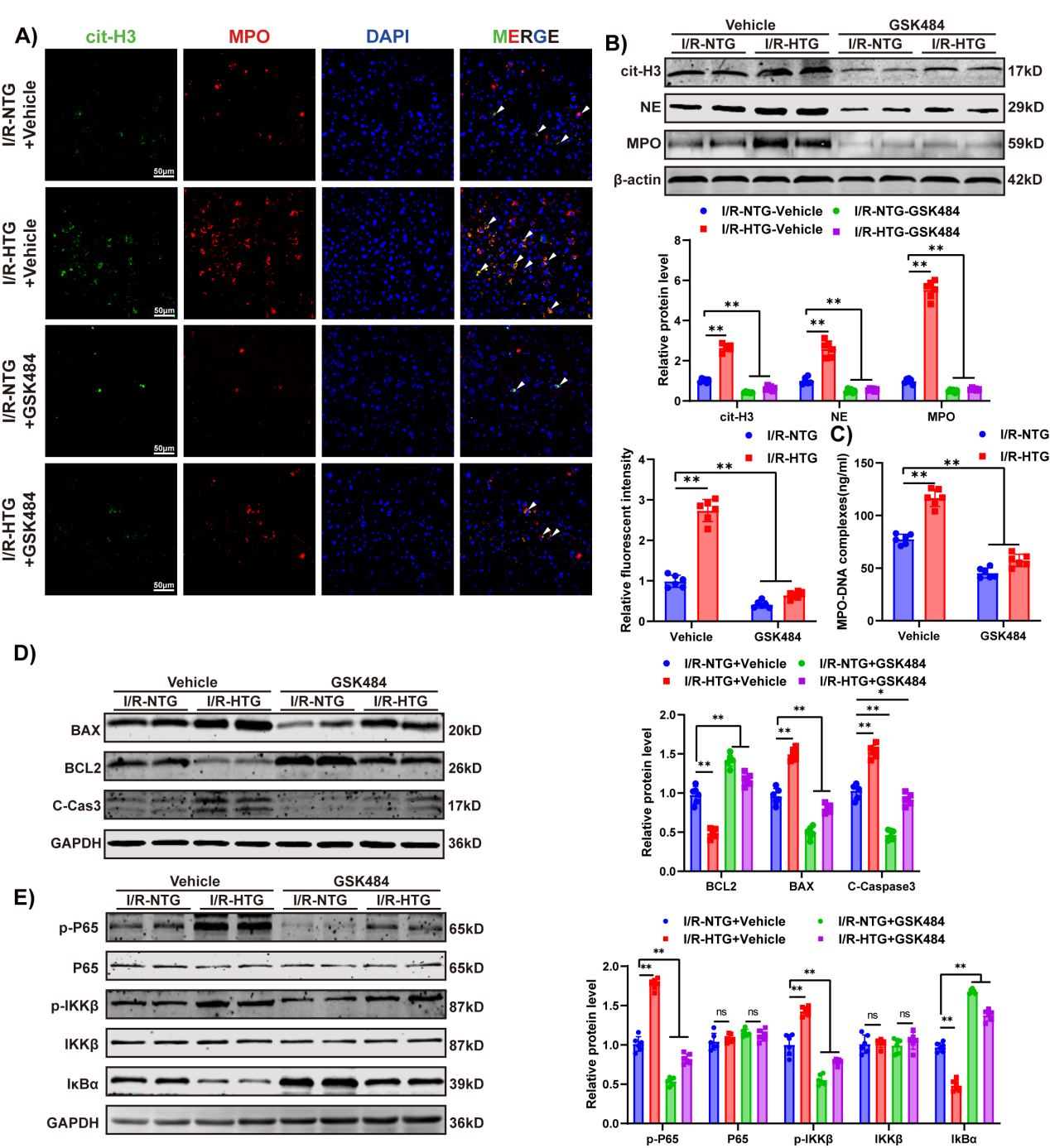


**Supplementary Figure 6. The PAD4 inhibitor GSK484 alleviates inflammation, apoptosis, and NETosis induced by RGS16 overexpression.**
(A) *Rgs16*-NTG and *Rgs16*-HTG mice received vehicle or GSK484 intraperitoneally and were then subjected to I/R. Representative immunofluorescence images of cit-H3 and MPO in liver tissues from the indicated groups (n = 6). Scale bars = 50 μm. (B) Protein levels of NET markers in liver tissues from the indicated groups (n = 6). (C) Levels of MPO-DNA complexes in liver tissues from the indicated groups (n = 6). (D) Protein levels of BCL2, BAX, and cleaved caspase-3 in liver tissues from the indicated groups (n = 6). (E) Protein levels of NF-κB signaling pathway molecules in liver tissues from the indicated groups (n = 6). Protein levels were normalized to GAPDH where applicable. Data are presented as mean ± SD and are representative of three independent experiments. Two-way ANOVA followed by Tukey’s multiple comparison test was used for comparisons among the vehicle- and GSK484-treated *Rgs16*-NTG and *Rgs16*-HTG groups. *P < 0.05, **P < 0.01; n.s., not significant.


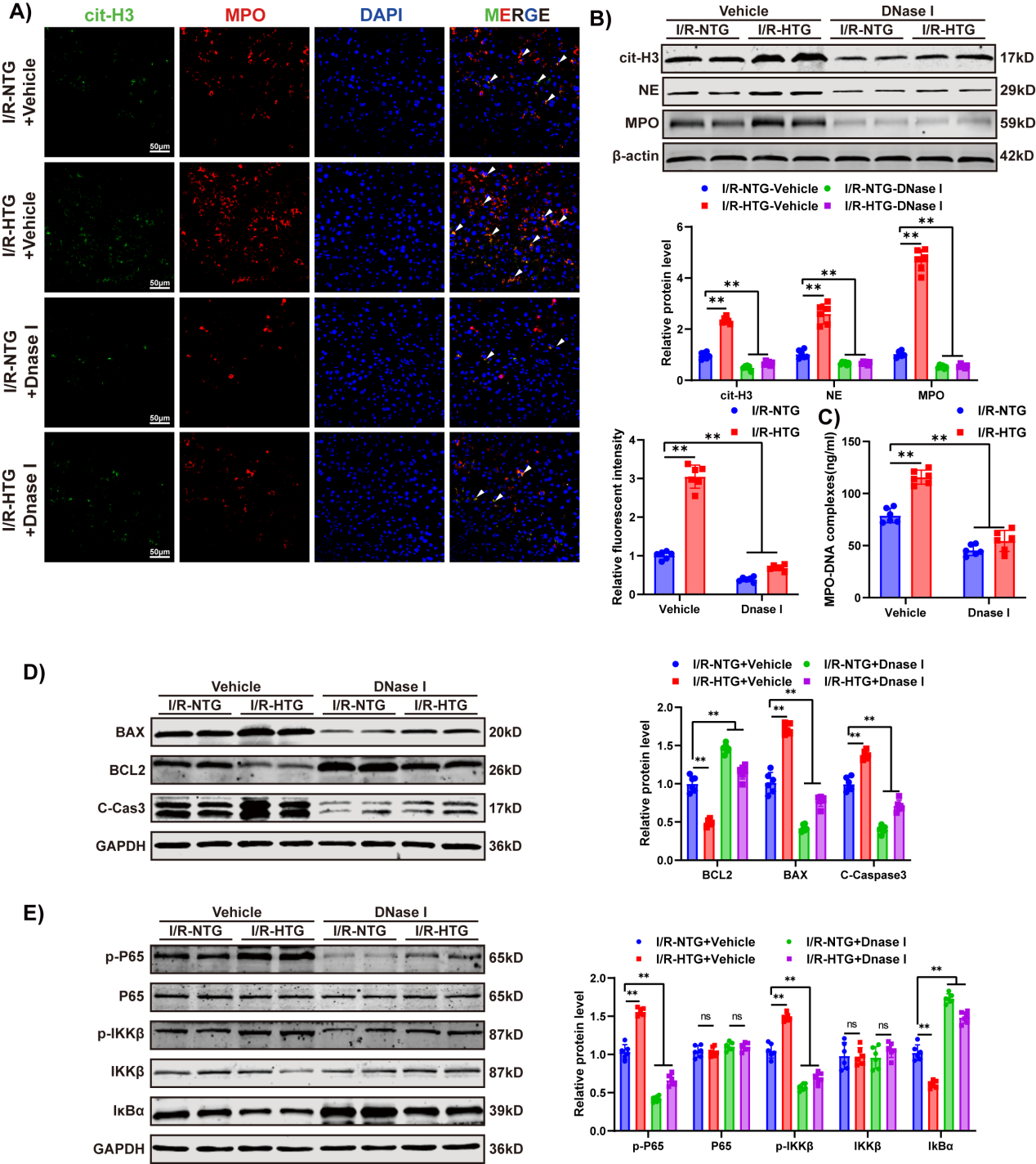


**Supplementary Figure 7. The NETosis inhibitor Dnase I alleviates inflammation, apoptosis, and NETosis induced by RGS16 overexpression.**

(A) *Rgs16*-NTG and *Rgs16*-HTG mice received vehicle or DNase I intraperitoneally and were then subjected to I/R. Representative immunofluorescence images of cit-H3 and MPO in liver tissues from the indicated groups (n = 6). Scale bars = 50 μm. (B) Protein levels of NET markers in liver tissues from the indicated groups (n = 6). (C) Levels of MPO-DNA complexes in liver tissues from the indicated groups (n = 6). (D) Protein levels of BCL2, BAX, and cleaved caspase-3 in liver tissues from the indicated groups (n = 6). (E) Protein levels of NF-κB signaling pathway molecules in liver tissues from the indicated groups (n = 6). Protein levels were normalized to GAPDH where applicable. Data are presented as mean ± SD and are representative of three independent experiments. Two-way ANOVA followed by Tukey’s multiple comparison test was used for comparisons among the vehicle- and DNase I-treated *Rgs16*-NTG and *Rgs16*-HTG groups. *P < 0.05, **P < 0.01; n.s., not significant.


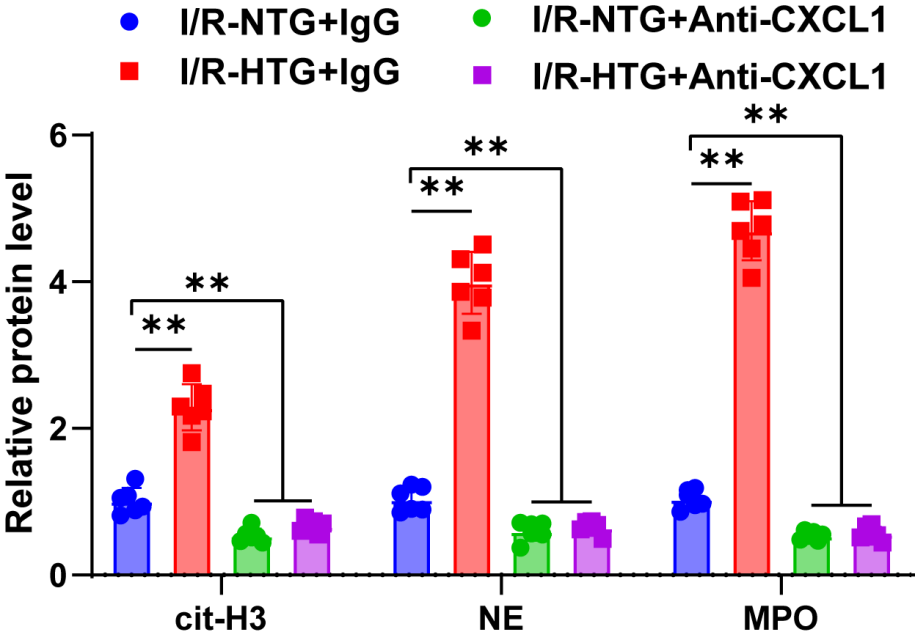


**Supplementary Figure 8. Quantification of NETs marker protein levels from *in vivo* neutralizing antibody experiments by Western blot.** Two-way ANOVA followed by Tukey’s multiple comparison test was used for comparisons among the IgG- and anti-CXCL1-treated *Rgs16*-NTG and *Rgs16*-HTG groups. **P < 0.01; n.s., not significant.

**
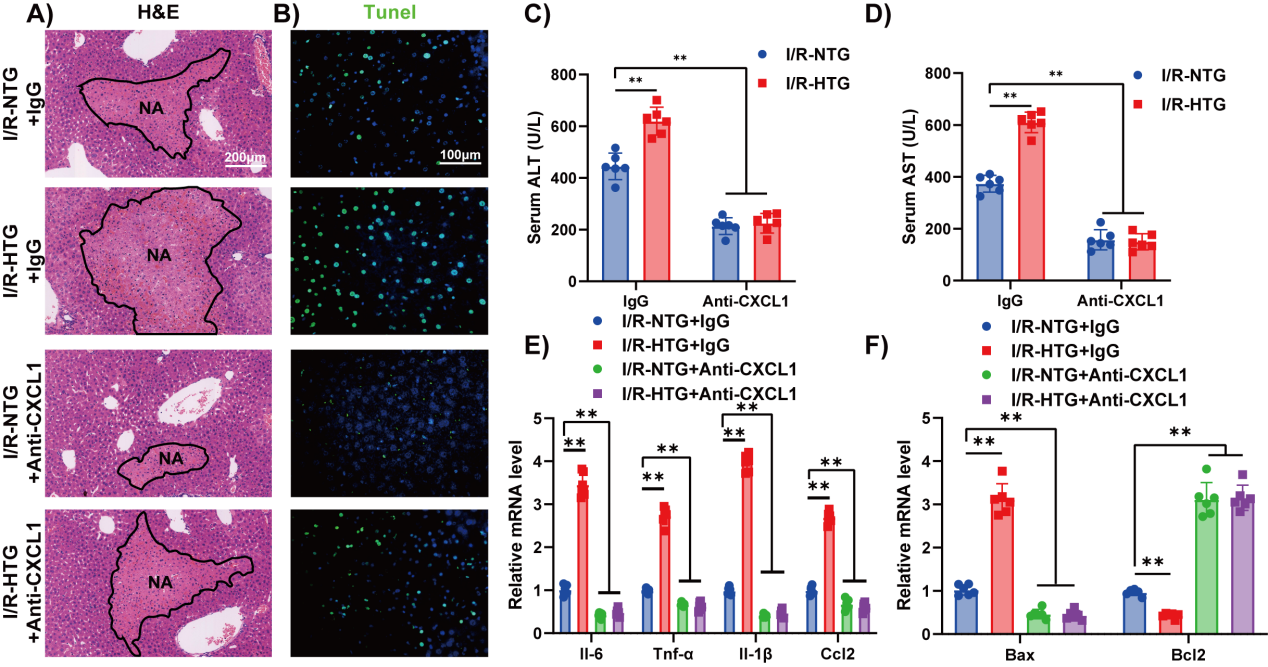
**

**Supplementary Figure 9. The anti-CXCL1 neutralizing antibodies alleviates inflammation and apoptosis induced by RGS16 overexpression.**
(A) *Rgs16*-NTG and *Rgs16*-HTG mice received IgG or anti-CXCL1 neutralizing antibody intraperitoneally and were then subjected to I/R. H&E staining of liver sections from the indicated groups (n = 6). Scale bar = 200 μm. NA, necrotic area. (B) TUNEL staining of liver sections from the indicated groups (n = 6). Scale bar = 100 μm. (C) Serum ALT levels in mice from the indicated groups (n = 6). (D) Serum AST levels in mice from the indicated groups (n = 6). (E) Relative mRNA expression levels of proinflammatory cytokine/chemokine genes in liver tissues from the indicated groups (n = 6). (F) Relative mRNA expression levels of Bcl2 and Bax in liver tissues from the indicated groups (n = 6). mRNA levels were normalized to GAPDH. Data are presented as mean ± SD and are representative of three independent experiments. Two-way ANOVA followed by Tukey’s multiple comparison test was used for comparisons among the IgG- and anti-CXCL1-treated *Rgs16*-NTG and *Rgs16*-HTG groups. **P < 0.01; n.s., not significant.


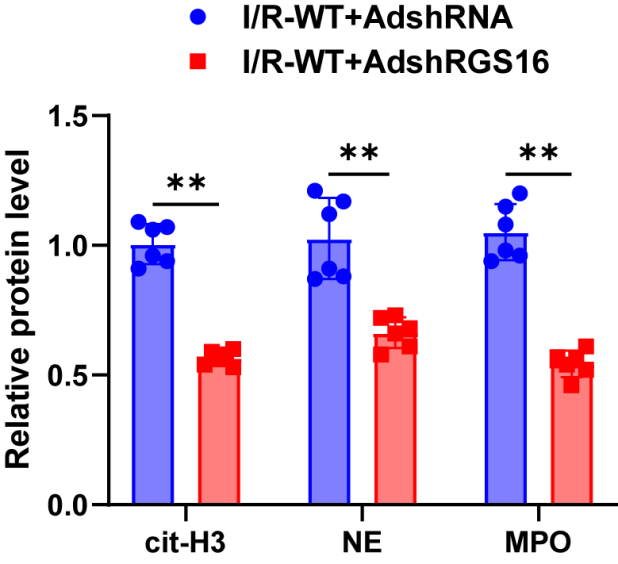


**Supplementary Figure 10. Quantification of NETs marker protein levels from *in vivo* knock-down adenovirus experiments by Western blot.** Two-tailed unpaired Student’s t-test was used for comparisons between AdshRNA- and AdshRGS16-treated groups. **P < 0.01; n.s., not significant.


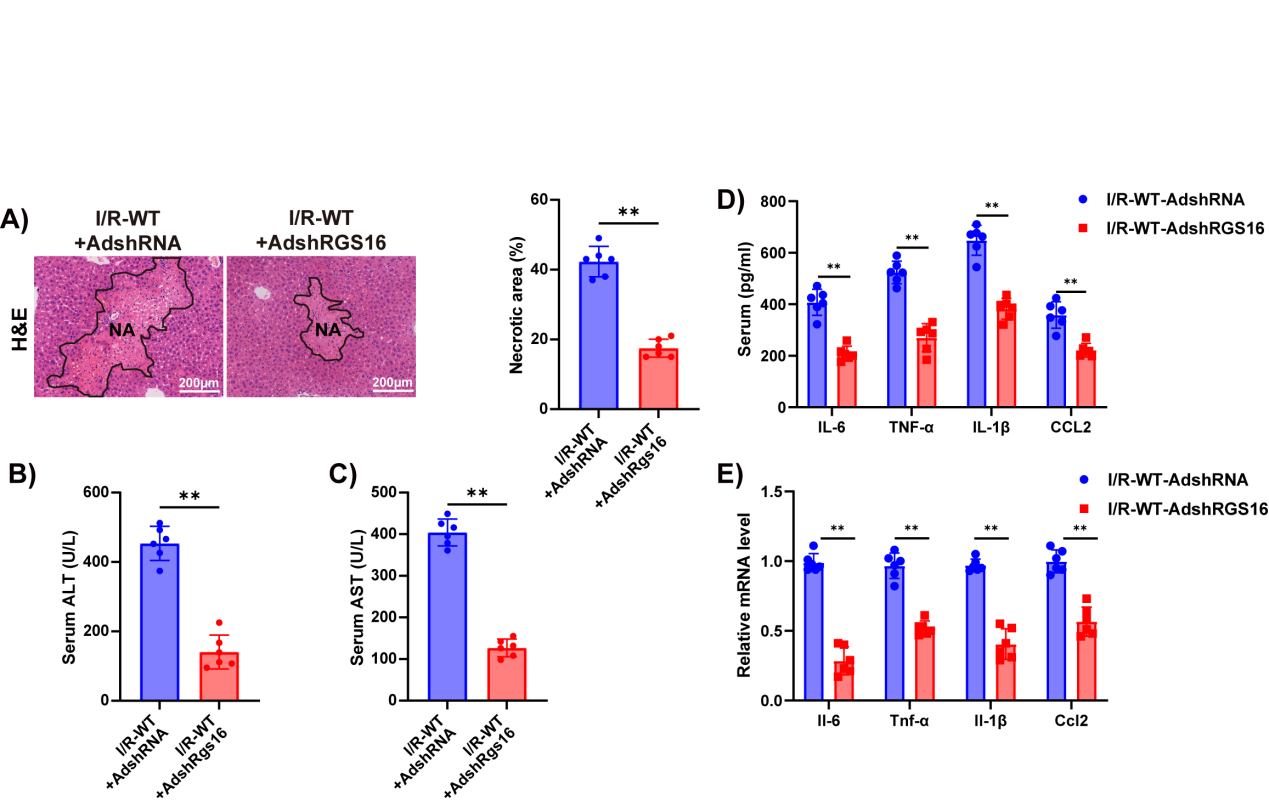


**Supplementary Figure 11. The RGS16 knockdown alleviates inflammation and apoptosis during HIRI *in vivo*.**
(A) WT mice received AdshRNA or AdshRGS16 intravenously and were then subjected to I/R. H&E staining of liver sections from the indicated groups (n = 6). Scale bar = 200 μm. (B) Serum ALT levels in mice from the indicated groups (n = 6). (C) Serum AST levels in mice from the indicated groups (n = 6). (D) Relative mRNA expression levels of proinflammatory cytokine/chemokine genes in liver tissues from the indicated groups (n = 6). (E) Relative mRNA expression levels of Bcl2 and Bax in liver tissues from the indicated groups (n = 6). mRNA levels were normalized to GAPDH. Data are presented as mean ± SD and are representative of three independent experiments. Two-tailed unpaired Student’s t-test was used for comparisons between AdshRNA- and AdshRGS16-treated groups. **P < 0.01; n.s., not significant.


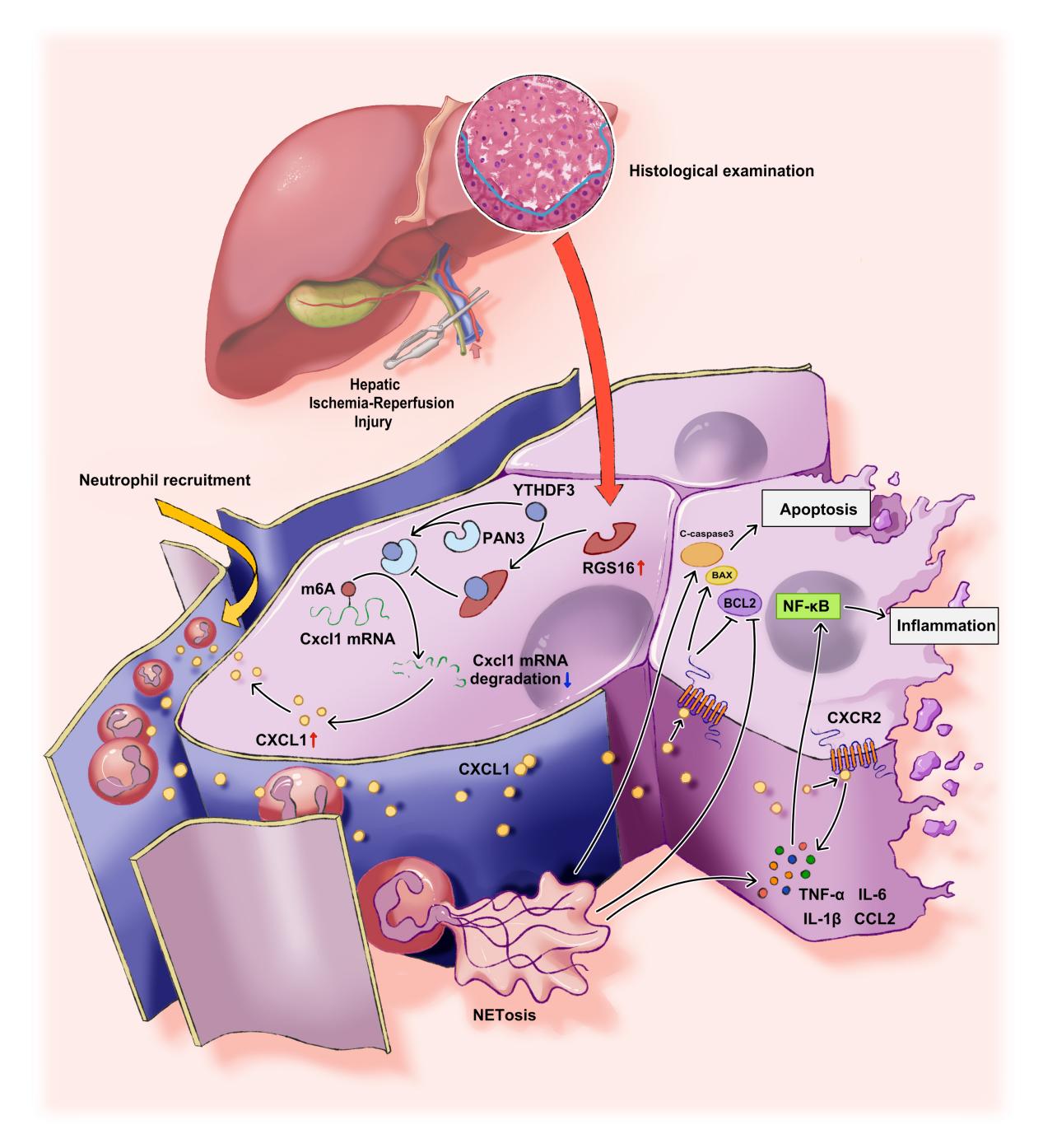


**Supplementary Figure 12. The general schematic diagram.**

**Supplementary Tables**

**Supplementary Table 1. The baseline clinical and operative characteristics of the patients.**

| No. | Sex | Diagnosis | Age | Preoperative Hemoglobin (g/L) | Preoperative platelet count (10^9/L) | Preoperative neutrophil count (10^9/L) | Preoperative total bilirubin  (μmol/L) | Preoperative direct bilirubin  (μmol/L) | Preoperative indirect bilirubin (μmol/L) | Ischemia method | Number of vascular occlusions | Intraoperative blood loss (ml) | Operation time (min) |
| --- | --- | --- | --- | --- | --- | --- | --- | --- | --- | --- | --- | --- | --- |
| 1 | F | Hemangioma | 63 | 135 | 259 | 2.94 | 17.8 | 6 | 11.8 | Pringle maneuver | 1 | 150 | 192 |
| 2 | F | Hemangioma | 62 | 131 | 263 | 2.85 | 10.9 | 3.7 | 7.2 | Pringle maneuver | 1 | 40 | 64 |
| 3 | F | Hemangioma | 56 | 147 | 246 | 3.49 | 17.8 | 5.9 | 11.9 | Pringle maneuver | 1 | 135 | 34 |
| 4 | F | Hemangioma | 64 | 123 | 245 | 2.1 | 10.5 | 2.7 | 8.4 | Pringle maneuver | 1 | 85 | 110 |
| 5 | F | Hemangioma | 47 | 138 | 261 | 3.06 | 6.4 | 1.6 | 4.7 | Pringle maneuver | 1 | 50 | 55 |
| 6 | F | Hemangioma | 52 | 130 | 280 | 2.91 | 8.3 | 2.8 | 5.5 | Pringle maneuver | 1 | 60 | 65 |
| 7 | M | Hemangioma | 39 | 120 | 282 | 2.8 | 8.7 | 2.2 | 6.1 | Pringle maneuver | 1 | 200 | 95 |
| 8 | F | Hemangioma | 44 | 133 | 219 | 2.78 | 7.3 | 2.4 | 4.9 | Pringle maneuver | 1 | 65 | 67 |
| 9 | F | Hemangioma | 49 | 117 | 276 | 2.67 | 17.8 | 4.5 | 13.2 | Pringle maneuver | 1 | 125 | 83 |
| 10 | F | Hemangioma | 59 | 118 | 259 | 2.6 | 18.7 | 6.2 | 12.8 | Pringle maneuver | 1 | 30 | 115 |
| 11 | M | Hemangioma | 37 | 149 | 208 | 2.73 | 15.3 | 3.8 | 11.8 | Pringle maneuver | 1 | 140 | 87 |
| 12 | F | Hemangioma | 47 | 136 | 212 | 3.74 | 7.9 | 1.8 | 6.1 | Pringle maneuver | 1 | 110 | 80 |
| 13 | F | Hemangioma | 51 | 131 | 237 | 3.53 | 6.9 | 2.3 | 4.6 | Pringle maneuver | 1 | 200 | 88 |
| 14 | F | Hemangioma | 34 | 142 | 251 | 2.44 | 9 | 3.4 | 6.6 | Pringle maneuver | 1 | 300 | 162 |
| 15 | F | Hemangioma | 42 | 133 | 193 | 1.71 | 9.7 | 2.6 | 6.5 | Pringle maneuver | 1 | 50 | 71 |
| 16 | M | Hemangioma | 53 | 113 | 225 | 5.09 | 29.3 | 7.6 | 21.7 | Pringle maneuver | 1 | 50 | 103 |
| 17 | M | Hemangioma | 47 | 143 | 178 | 2.76 | 12.8 | 5 | 7.8 | Pringle maneuver | 1 | 90 | 77 |
| 18 | F | Hemangioma | 38 | 131 | 280 | 2.98 | 8.6 | 2.2 | 6.4 | Pringle maneuver | 1 | 55 | 91 |
| 19 | M | Hemangioma | 47 | 160 | 187 | 2.4 | 11.2 | 3.3 | 7.9 | Pringle maneuver | 1 | 95 | 70 |
| 20 | M | Hemangioma | 35 | 134 | 279 | 3.1 | 15 | 4.5 | 10.5 | Pringle maneuver | 1 | 90 | 65 |
| 21 | M | Hemangioma | 53 | 166 | 365 | 4 | 11.2 | 3.2 | 8 | Pringle maneuver | 1 | 30 | 122 |
| 22 | F | Hemangioma | 44 | 121 | 299 | 4.21 | 14.4 | 3.7 | 10.7 | Pringle maneuver | 1 | 70 | 195 |
| 23 | F | Hemangioma | 60 | 152 | 222 | 1.94 | 11.5 | 4.6 | 6.9 | Pringle maneuver | 1 | 85 | 120 |
| 24 | F | Hemangioma | 40 | 140 | 244 | 3.11 | 13.1 | 3.8 | 9.3 | Pringle maneuver | 1 | 60 | 84 |
| 25 | F | Hemangioma | 53 | 123 | 344 | 3.69 | 6.6 | 2.1 | 4.5 | Pringle maneuver | 1 | 50 | 125 |
| 26 | F | Hemangioma | 67 | 127 | 226 | 3.8 | 11 | 2.8 | 8.2 | Pringle maneuver | 1 | 50 | 96 |
| 27 | F | Hemangioma | 50 | 130 | 201 | 3.93 | 15.9 | 4.9 | 11 | Pringle maneuver | 1 | 20 | 78 |
| 28 | M | Hemangioma | 45 | 138 | 250 | 3.17 | 12.5 | 3.5 | 9 | Pringle maneuver | 1 | 70 | 115 |
| 29 | F | Hemangioma | 40 | 150 | 245 | 1.65 | 8.2 | 1.7 | 6.5 | Pringle maneuver | 1 | 130 | 80 |
| 30 | F | Hemangioma | 58 | 158 | 231 | 1.9 | 21.8 | 6.6 | 15.2 | Pringle maneuver | 1 | 40 | 92 |
| 31 | F | Hemangioma | 60 | 119 | 218 | 1.98 | 13.5 | 3.6 | 9.9 | Pringle maneuver | 1 | 100 | 100 |
| 32 | F | Hemangioma | 49 | 127 | 241 | 2.2 | 11.2 | 3.1 | 8.1 | Pringle maneuver | 1 | 85 | 88 |
| 33 | F | Hemangioma | 42 | 137 | 205 | 2.17 | 20.4 | 6.4 | 14 | Pringle maneuver | 1 | 60 | 90 |
| 34 | F | Hemangioma | 47 | 142 | 237 | 2.09 | 15.7 | 3.7 | 12.7 | Pringle maneuver | 2 | 200 | 435 |
| 35 | F | Hemangioma | 66 | 119 | 241 | 3.11 | 9.3 | 2.8 | 6.5 | Pringle maneuver | 2 | 125 | 240 |
| 36 | F | Hemangioma | 56 | 129 | 196 | 2.24 | 10.7 | 3.4 | 7.3 | Pringle maneuver | 2 | 110 | 120 |
| 37 | F | Hemangioma | 52 | 141 | 322 | 2.45 | 12 | 2.2 | 9.8 | Pringle maneuver | 2 | 80 | 165 |
| 38 | F | Hemangioma | 37 | 128 | 171 | 3.44 | 19.3 | 3.8 | 15.1 | Pringle maneuver | 2 | 90 | 135 |
| 39 | M | Hemangioma | 56 | 122 | 318 | 2.82 | 20.8 | 6.3 | 14.9 | Pringle maneuver | 2 | 175 | 166 |
| 40 | F | Hemangioma | 25 | 125 | 282 | 2.68 | 17.9 | 3.8 | 14.1 | Pringle maneuver | 2 | 200 | 130 |
| 41 | F | Hemangioma | 37 | 122 | 214 | 4.36 | 17.3 | 5.5 | 12.2 | Pringle maneuver | 2 | 115 | 135 |
| 42 | F | Hemangioma | 51 | 148 | 327 | 3.1 | 7.2 | 2.2 | 5.1 | Pringle maneuver | 2 | 110 | 145 |
| 43 | F | Hemangioma | 38 | 135 | 261 | 2.05 | 4 | 1 | 3 | Pringle maneuver | 2 | 180 | 190 |
| 44 | M | Hemangioma | 42 | 167 | 235 | 1.98 | 8.8 | 2.4 | 6.4 | Pringle maneuver | 2 | 220 | 225 |
| 45 | F | Hemangioma | 61 | 130 | 258 | 2.4 | 7.9 | 2.6 | 5.3 | Pringle maneuver | 2 | 110 | 135 |
| 46 | F | Hemangioma | 50 | 155 | 230 | 3.22 | 25.2 | 6.7 | 18.5 | Pringle maneuver | 2 | 150 | 112 |
| 47 | F | Hemangioma | 60 | 126 | 349 | 3.69 | 7.6 | 1.4 | 6.2 | Pringle maneuver | 2 | 120 | 90 |
| 48 | F | Hemangioma | 53 | 120 | 171 | 2.61 | 10.9 | 2.8 | 8.1 | Pringle maneuver | 2 | 250 | 220 |
| 49 | F | Hemangioma | 40 | 144 | 205 | 2.52 | 10.8 | 3.3 | 7.5 | Pringle maneuver | 2 | 280 | 180 |
| 50 | F | Hemangioma | 47 | 118 | 365 | 2.15 | 8.6 | 2.4 | 6.2 | Pringle maneuver | 3 | 210 | 305 |
| 51 | F | Hemangioma | 59 | 117 | 250 | 1.78 | 14.4 | 3.3 | 11.1 | Pringle maneuver | 3 | 160 | 235 |
| 52 | M | Hemangioma | 33 | 154 | 191 | 2.14 | 7 | 1.5 | 5.5 | Pringle maneuver | 3 | 260 | 165 |
| 53 | F | Hemangioma | 66 | 138 | 211 | 2.91 | 21.1 | 4.8 | 16.5 | Pringle maneuver | 3 | 170 | 155 |
| 54 | M | Hemangioma | 48 | 126 | 217 | 2.18 | 18.7 | 4.7 | 14 | Pringle maneuver | 3 | 280 | 165 |
| 55 | F | Hemangioma | 67 | 133 | 223 | 2.15 | 9.2 | 2.6 | 6.7 | Pringle maneuver | 3 | 100 | 160 |
| 56 | F | Hemangioma | 40 | 128 | 194 | 2.83 | 12.5 | 3 | 9.9 | Pringle maneuver | 3 | 150 | 170 |
| 57 | F | Hemangioma | 35 | 142 | 247 | 2.96 | 13.6 | 3.3 | 10.1 | Pringle maneuver | 3 | 400 | 199 |
| 58 | F | Hemangioma | 51 | 151 | 199 | 3.67 | 10.3 | 5 | 7.5 | Pringle maneuver | 3 | 235 | 175 |
| 59 | F | Hemangioma | 61 | 114 | 204 | 1.81 | 12.9 | 2.7 | 10.2 | Pringle maneuver | 3 | 225 | 165 |
| 60 | F | Hemangioma | 57 | 146 | 217 | 1.97 | 12.2 | 4 | 9.2 | Pringle maneuver | 4 | 180 | 255 |

**Supplementary Table 2. shRNA&siRNA target sequence.**

| Gene name Sequence ( 5’- 3’) | |
| --- | --- |
| shRgs16-1  shRgs16-2  shRgs16-3  siYTHDF3  siCXCR2-1  siCXCR2-2  siCXCR2-3 | CCGAGAACTGACCAAGACAAA  TGGGCCAGTAAGCATAACAAA  GAGTTCAAGAAGATCCGATCA  TGGCAATGATACTTTGAGTAAGG  AAGAAUCUCCGUAGCAUUCAA  UGUAUUGUUACCUACAUCCTT  UUCAAUGUUGAACUUAUCCTT |

**Supplementary Table 3. Primer sequence.**

| Gene name Sequence ( 5’- 3’) | |
| --- | --- |
| **Homo sapiens：**  RGS16-F  RGS16-R  GAPDH-F  GAPDH-R  **Mus musculus:**  Rgs16-F  Rgs16-R  Tnf-α-F  Tnf-α-R  Il-6-F  Il-6-R  Il-1β-F  Il-1β-R  Ccl2-F  Ccl2-R  Cxcl1-F  Cxcl1-R  Bax-F  Bax-R  Bcl2-F  Bcl2-R  Cxcr2-F  Cxcr2-R  Ythdf3-F  Ythdf3-R  Gapdh-F  Gapdh-R | ATCAGAGCTGGGCTGCGATA  CAGGTCGAACGACTCTCTCC  GGAGCGAGATCCCTCCAAAAT  GGCTGTTGTCATACTTCTCATGG  TTGACGAGTACATCCGCAGC  TCTCCATCAATGTGCGGGTC  CCCTCACACTCAGATCATCTTCT  GCTACGACGTGGGCTACAG  TAGTCCTTCCTACCCCAATTTCC  TTGGTCCTTAGCCACTCCTTC  GCAACTGTTCCTGAACTCAACT  ATCTTTTGGGGTCCGTCAACT  CCAACCACCAGGCTACAGG  GCGTCACACTCAAGCTCTG  CTGGGATTCACCTCAAGAACATC  CAGGGTCAAGGCAAGCCTC  TGAGCGAGTGTCTCCGGCGAAT  GCACTTTAGTGCACAGGGCCTTG  TGGTGGACAACATCGCCCTGTG  GGTCGCATGCTGGGGCCATATA  ATGCCCTCTATTCTGCCAGAT  GTGCTCCGGTTGTATAAGATGAC  GATCAGCCTATGCCATATCTGAC  CCCCTGGTTGACTAAAAACACC  AAGAGGGATGCTGCCCTTAC  AATCCGTTCACACCGACCTT |

**Supplementary Table 4. MeRIP-qPCR and RIP-qPCR Primer.**

| Gene name Sequence ( 5’- 3’) | |
| --- | --- |
| Cxcl1-F  Cxcl1-R | TTTGTGTCTAGTTGGTAGGGCATA  CATTTGTAACAGTCCTTTGAACG |

**Supplementary Table 5. Antibody Information.**

| Antibody Company Cat No. Dilution | | | |
| --- | --- | --- | --- |
| RGS16  PAN3  cit-H3  MPO  IκBa  Phospho- p65  p65  Phospho-IKKB  IKKB  BAX  BCL-2  c-Caspase3  Ly6G  YTHDF3  CXCL1  MPO  NE  FLAG  His  HA  GAPDH  Beta actin | Santa Cruz  Santa Cruz  Abcam  R&D systems  Cell Signaling Technology  Cell Signaling Technology  Cell Signaling Technology  Cell Signaling Technology  Cell Signaling Technology  Cell Signaling Technology  Cell Signaling Technology  Cell Signaling Technology  Cell Signaling Technology  Proteintech  Proteintech  Proteintech  Proteintech  Proteintech  Proteintech  Proteintech  Proteintech  Proteintech | sc-166083  sc-376434  ab281584  AF3667  4812  3033  4764  2697  8943  2772  3498  9664  88876  25537-1-AP  12335-1-AP  22225-1-AP  27642-1-AP  66008-4-Ig  66005-1-Ig  51064-2-AP  60004-1-Ig  66009-1-Ig | 1:1000 (WB);1:100 (IHC)  1:1000 (WB)  1:1000 (WB);1:100 (IF)  1:100 (IF)  1:1000 (WB)  1:1000 (WB)  1:1000 (WB)  1:1000 (WB)  1:1000 (WB)  1:1000 (WB)  1:1000 (WB)  1:500 (WB)  1:200 (IF)  1:1000 (WB)  1:1000 (WB);1:100 (IHC)  1:500 (WB)  1:500 (WB)  1:1000 (WB)  1:1000 (WB)  1:1000 (WB)  1:50000 (WB)  1:20000 (WB) |
